# Digital Footprint and Its Impact on Improving Customer Experience: An Exploratory Study of a Sample of Internet Companies in Nineveh Governorate

**DOI:** 10.12688/f1000research.174437.1

**Published:** 2026-01-24

**Authors:** Abdul Sattar Salim Awadh Al – Jubory, Saif Khalid Zakaria, Mohammad Mahmood Al-Mulla Hasan

**Affiliations:** 1 Business Administration, University of Kirkuk, Kirkuk, Kirkuk Governorate, Iraq; 2 Management Information Systems, University of Mosul, Mosul, Nineveh Governorate, Iraq; 3 Marketing Management, University of Mosul, Mosul, Nineveh Governorate, Iraq

**Keywords:** Digital Footprint, Customer Experience, Personalization, Internet Service Providers, Nineveh.

## Abstract

**Background:**

The current study attempts to fill a significant and crucial deficiency in knowing how to trigger the digital footprints for enhancing service quality from the customers’ point of view among Internet service providers as well, especially under the Iraqi environment, Mosul city, Nineveh Governorate. The sector is in a unique competitive situation, and although this strategic function of digital data is extremely critical for personalization initiatives as well as for competitive advantage, very little practical use has been made in such an environment. Indeed, metrics such as (data governance maturity and customer trust) emerged as challenges impacting the use of data analytics and their integration into the improvement of customer experience. As such, this study seeks to understand how local Internet service providers (ISPs) can be empowered by individual customers’ digital footprints, evaluate what influences their most effective use in order to enhance customer experience, and discover best explorations for guidance on appropriate and ethical strategies.

**Methodology:**

The method data were collected from (ISPs) using a structured questionnaire, issued to a representative sample of ISP and overall number of (319).

**Results:**

However, the results obtained using statistical analysis indicate that while digital footprints significantly enhance service personalization and support efficiency, there is a substantial implementation gap between data collection and its application to design value-adding interactions. This, in turn, suggests a need to strengthen the analysis capacity of service providers.

**Conclusion:**

This paper ends with an insight; for Internet service providers to facilitate customer digital footprints and the digital data left by customers to enhance the quality of services, they must adopt a balanced, evidence-based strategy as well as transparent consideration of privacy concerns that sustain user trust.

## Introduction

Given the pace of technology uplift, companies were willing to adopt more radical change in pursuit of the goal of becoming fluid organizations and customer-centric ones that would allow them to better control the customer experience across increasingly complex markets that connect with one another. All of this has combined to make customer experience (CX) a key differentiator ( Lemon & Verhoef, 2016). On the other hand, the services on the Internet are experiencing a notable radical change, especially in the emerging context where the customer has to interact online with Internet service providers using electronic channels and modern devices such as smart phones. For example, we can mention some of the data-driven differentiators that, in turn, have forced a number of firms to pursue customer orientation by channelling power from digital footprints to differentiate themselves. Furthermore, people’s virtual-digital footsteps are more precious than anything ( Hassan, 2024).

From the foregoing, the concept of digital footprints has evolved: the trace of user data when users are online. It is dynamic and constantly updated, and is a medium for predicting behavior or preference, making it useful for business ( Neelam et al., 2025). ( Koidl et al., 2018) found that it is possible to systematically retrieve and analyze digital traces, so companies know their customers in detail, predict hidden needs for them, and make personalized offers and proactive support available. The digitization of information and data capture has become an increasingly appealing concern for companies seeking to foster a better customer experience, although it is far from free of ethical and strategic conflict ( Wedel & Kannan, 2016). On the other hand, opaque data practices could challenge faith, pose privacy risks, and make customers feel that they are being watched too much, which needs a balance ( Martin & Murphy, 2016). Furthermore, the potential of digital footprints varies depending on several parameters, such as transparent governance, responsible data treatment, and customer awareness of the collection and usage of their data ( Acquisti et al., 2015). However, despite the increasing content on digital personalization and data related to consumers ( Wedel & Kannan, 2016), companies should train existing employees in data analysis and hire new staff with such capabilities because it is crucial in the near future. However, research on how digital footprints specifically contribute to improving customer experience in Internet service markets is still scarce, while comparing this process between developing contexts and mature ones, where the services, digital culture, and customers’ minds vary, is contextual.

In addition, the extent to which Internet Service Providers (ISPs) intentionally use digital footprints to shape and improve customer journeys has not been sufficiently studied in local environments.

Based on this, the aim of this study is to explore the role of digital footprints in improving customer experience within Internet service companies in Nineveh Governorate, Iraq, through

1. Examining current data usage practices.
2. Levels of organizational awareness and customer awareness.
3. The perceived outcomes associated with digital footprint–based personalization.

This study seeks to fill a critical gap in the literature and provide evidence-based insights to enhance innovation in the service sector in markets that experience digital transformation.

## 1. Literature review

A literature review is also important for situating the current study from previous research studies and describing the research gap more precisely. To this effect, ( Dhal et al., 2025) pointed out that technological progress has also had a positive impact on customer experience enhancement. The ubiquitous use and evolution of digital platforms have allowed businesses to accumulate and analyze digital footprints to enhance customer experience, particularly in the flourishing service industry ( Arya et al., 2019).

On the other hand, ( Micheli et al., 2018) highlighted that these digital footprints represent one of the dimensions of digital inequality, considering all the data left by users that can be exploited to customize services and hence improve user experience. Some people own their data, and some do not, and companies that use these digital trails in the right way will make enormous economic and marketing gains.

Conversely, the research by ( Martin & Murphy, 2016) uncovered a series of key issues around privacy perceptions, data transparency, and trust in relation to the use of customers’ digital foot research, showing that personalized services improve the quality of experience, yet too much or non-transparent data tracking can result in concerns, thus negatively affecting customers’ attitudes. Moreover, digital trace advantages are not uniformly available among social groups and individuals as there is variation in digital culture. Different levels of internet use may affect the degree to which various relationships can be improved or experience difficulties ( Micheli et al., 2018).

We conclude from the above that there is a growing interest in the concept of digital footprint, but empirical evidence in developing contexts is limited, particularly regarding how Internet service providers (ISPs) use digital footprints to improve customer experience in areas such as Nineveh Governorate, as the level of digital adoption and customer awareness varies from region to region. This study seeks to address this gap by examining how ISPs in Nineveh use digital footprint data to enhance the customer experience.

### 1.1 Study problem

Previous research and literature review also pointed towards the ambivalence of digital footprints: it can be an a driver of personalized customer experiences on the one hand, and a possible cause on the one hand, and via privacy and trust concerns on the other ( Martin & Murphy, 2016). ( Golder & Macy, 2014) also observed that digital traces present unprecedented and exciting research opportunities because of the impossibility of generalizing the findings and the changing character of data over time. Furthermore, evidence is limited and ambiguous in the ISP industry (Martin & Murphy, 2017).

Background Prior to this work, no studies have assessed how ISPs operate within Nineveh Governorate, Iraq especially exploit digital customer footprints and how they are influencing, enhancing and tailoring the customer journey or how this approach is received [i.e., cognitive and behavioral responses to such data-driven strategies]. Hence, there is contextual research with respect to the mechanisms in which digital traces can be used to improve customer experience in this local service environment and their strategic use. Therefore, the research problem can be described succinctly as:

- How are traces left in the digital environment enhancing customer experience on Internet Mosul service providers and how are these effects produced?

### 1.2 The importance of the study

The relevance of this research focuses on the attributes involved which leads to:

1. Understanding customers and their needs, preferences, and expectations through analysis of their digital footprint and how they engage with digital channels.
2. Facilitating the provision of customized customer experiences helps improve overall client satisfaction and long-term loyalty.
3. This adds to the competitive edge for companies that make efficient use of digital footprint analysis in particular types of markets that are adaptive.

### 1.3 Study objectives

1. Identify best practices for employing a customer’s digital footprint to enhance the customer experience.
2. Assess the impact of using a customer’s digital footprint on improving customer experiences.
3. Provide recommendations to businesses on how to effectively use a customer’s digital footprint.

## 2. Conceptual framework

### 2.1 Digital footprint

In Arabic, the verb “ *basama*” refers to imprinting or marking with the fingertip, and the term “ *basmah*” denotes the trace left by the finger. The term originally referred to the unique imprint that distinguishes one individual from another, similar to the uniqueness of the human fingerprint, voiceprint, retinal pattern, ear shape, and other biometric identifiers that do not replicate or match among individuals ( Ibn Manzur, 1998).

The concept of *digital footprint* first emerged under the expression “the snail trail,” as mentioned by ( Negroponte, 2015), and was later termed “data remnants” by Tim O’Reilly. Initially, it referred only to traces of information left behind after browsing the internet. Today, however, the term digital footprint or digital shadow refers to the data generated and used, regardless of the type of device involved ( Kligienė, 2012).

According to ( Majeed et al., 2020), a Digital Footprint can be described as a digital file or shadow that constantly follows the user, such as a tattoo that cannot be easily erased as it continues to exist even after a person tries to remove it. ( Kligienė, 2012) further explains that the digital footprint represents the impact of an individual’s actions in the digital environment, including the use of mobile phones, television, the internet, or any other device equipped with sensors. This term is relevant not only to persons but also to organizations, as it describes a broad spectrum of online actions, communication, and behavior ( Golder & Macy, 2014). In line with ( Arya et al., 2019), digital footprints are the collection of data or information about an individual that is intentionally and unintentionally left behind, while being active on the Internet, such as visiting websites or using online social networking sites. The desire to learn customer behavior in a digital economy and in a digital environment rather than in a traditional market leads to a substantial increase in interest in the study of digital footprint because Internet use is increasing ( Rauniar et al., 2013). The scope of digital footprint generation has also expanded significantly with the advent of the interactive web, which is expected to increase 44-fold by 2020 ( Arya et al., 2019). In addition, the expansion of mobile industries, 4G networks, and cloud computing has boosted the adoption of social media, which has led to greater adoption by consumers on smart devices and thus an increase in the size of digital footprints. Users constantly create digital footprints through comments, photos, videos, blogs, ratings, e-shopping, and interactions with the government and service apps. Studies conducted by ( Charlesworth, 2014) show that digital footprints reveal users’ interests, social identities, cultural backgrounds, professional affiliations, and geographical connections, making them particularly valuable for companies wishing to track customer behavior and build personal profiles for them. Thus, the digital footprint can be understood as user-related information within the digital environment.

### 2.2 Types of customer digital footprints

The literature and research indicate that customer Digital Footprints fall into two main categories: active and passive Digital Footprints.

1. Active Digital Footprints: These type of data refer to data that users intentionally create and share during their online interactions ( McDermot, 2018).
2. In contrast, negative Digital Footprints are generated without explicit user awareness as a result of background data collection embedded within websites, applications, and network infrastructures ( Micheli et al., 2018). These effects include Internet Protocol (IP) addresses, geolocation logs, device-specific metadata, browsing behavior, and platform-specific interaction patterns. These forms of invisible data capture are particularly common in targeted advertising systems ( Sörqvist & Holmgren, 2022). Recent research suggests that platforms are increasingly relying on negative behavioral data, not only to personalize digital experiences but also to influence decision-making processes (Raimondo et al., 2023).

As a result, the distinction between these two types of data has become central to discussions on privacy, data ethics, and customer autonomy in the digital marketplace ( Zuboff, 2019).

### 2.3 Advantages and disadvantages of customer digital footprints

Table 1 outlines the advantages and disadvantages of a customer’s Digital Footprint.

**Table 1.** Advantages and disadvantages of customer digital footprints.

Aspect	Advantages (Positive implications)	Disadvantages (Negative implications)	Source
**Personalization**	Enables companies to personalize content, recommendations, and marketing messages based on customer interests, improving relevance and engagement.	Excessive personalization may lead to a sense of surveillance and loss of autonomy, negatively affecting trust.	( Robles-Carrillo, 2024)
**User Experience**	Enhances customer experience by providing smoother navigation, tailored service offerings, and predictive support features.	The complexity and volume of digital trace data make it difficult for users to manage or control their digital identity over time.	( Hassan, 2024)
**Social Interaction**	Improves social connectivity by suggesting relevant communities, friends, and content based on shared interests across social networks.	Customer profiling can be used to infer sensitive personal attributes, raising ethical concerns about data misuse.	( Kligienė, 2012)
**Location-Based Services**	Enables accurate and efficient delivery of location-based services such as mapping, local recommendations, and service coverage optimization.	Passive and continuous collection of geolocation data may occur without the user’s explicit awareness or consent.	( Arya et al., 2019)

Source: By authors.

### 2.4 The concept of customer experience

Researchers define customer experience (CX) as the accumulated impressions of the customer across all points of direct and indirect interaction with the company and cover the pre-, during-, and post-consumption stages; that is, it is not a single event, but rather an interactive journey that includes perception, emotion, and cognitive evaluation as a result of the customer’s interaction with the brand through multiple channels ( Lemon & Verhoef, 2016). However ( Gahler et al., 2023) highlighted that customer experience is shaped by the functional, emotional, and social aspects of interaction that make it a personal response that varies from client to client based on individual expectations. while ( Hardcastle et al., 2025) explained that companies are increasingly focusing on designing integrated experiences across digital channels, which requires understanding customer needs, analyzing their data, and personalizing interactions to enhance the perceived value of the relationship. However ( Homburg et al., 2020) argued that customer experience is closely related to the level of customer engagement in interacting with the service provider, making it a collaborative process rather than a one-way process. We believe that it is a multidimensional response and is determined by the extent to which the organization manages its touchpoints and valuable experiences across the various digital channels of services.

### 2.5 Customer experience dimensions

Customer experience is a dual-direction, multi-layered process that scholars describe through several interconnected dimensions.

1. **Relational Dimension**: It relates to the quality of long-term relationships that customers and companies have with each other, which comprises elements such as trust, customization, and clear communication. All these factors are paramount for value-in-use and loyalty ( Ponsignon et al., 2017).
2. **Service, Physical environment**: Factors in this dimension are the usability and accessibility of service places and making digital and physical service touchpoints user-friendly. These determinants in combination affect customers’ cognitive appraisals and emotions during service encounters ( Dong et al., 2008).
3. **The social layer of the experience** of the behavior and action of other users is created in this layer when others influence and create a sense of belonging and how they can shape the emotional aspect too ( Siqueira Junior et al., 2025).
4. **Organizational Ability to Deliver Dependable and Responsive Service**: This relates to the firm’s customer service as well as the speed and accuracy of a company’s response to effectively solve problems that enhance satisfaction and increase repurchase ( Santos-Vijande et al., 2012).

In light of these perspectives, and based on the literature concerning digital footprints and customer experience, the conceptual framework of the present study was developed to reflect how digital footprints may operate across these dimensions to influence the overall customer experience.

**Figure 1.**
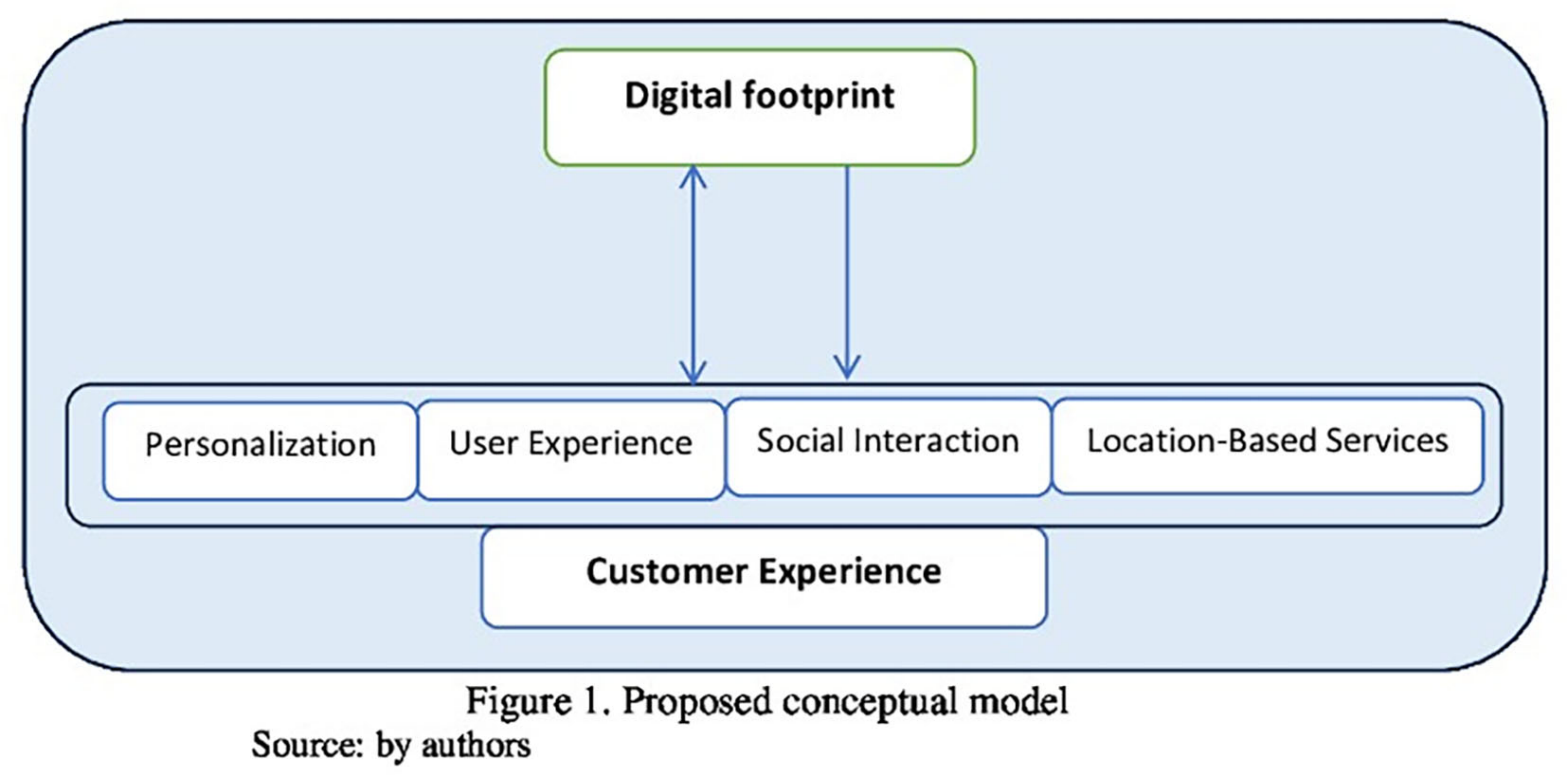
Conceptual model of the study. This figure presents the proposed conceptual model illustrating the relationship between digital footprints and customer experience in Internet service companies.

Based on the proposed model in Figure 1 and the rationale previously explained, we formulated the following hypotheses:

1. There were statistically significant differences in the respondents’ perceptions of the research variables (digital footprint and customer experience).
2. There is no statistically significant correlation between the digital footprint and customer experience among Internet service companies in Nineveh Governorate.
3. The digital footprint has no statistically significant effect on improving customer experience in Internet service companies in Nineveh Governorate *.*

## 3. Methods

**Research Population and Sample:** Internet service companies working in Nineveh Governorate were the research population as a result, three hundred and nineteen subjects who served as the sample were given the questionnaire. All respondents were adults, and online informed consent was obtained on the first page of the Web-based survey. Before answering the questionnaire, all participants clearly stated the purpose of the study and that their participation would be voluntary (meaning they could withdraw from the test any time); they were also assured that test information would remain confidential and not contain personal identification. A signature on a written consent form was not necessary because the data were collected in an online minimal risk survey that did not ask for any personal or sensitive information; thus, electronic consent is appropriate and adequate for this type of research as detailed in the Data Availability section.

**Statistical Methods Used:** The researchers used a set of statistical methods to analyze the data collected through the questionnaire, including frequencies, percentages, means, and standard deviations. Pearson’s correlation coefficient was employed to measure the strength of the relationship between the variables, and simple linear regression was used to measure the effect of the independent variables on the dependent variable.

## 4. Description and diagnosis of the research variables and testing of its hypotheses

**This section will include three paragraphs:**

### 4.1 Description of the research participants

Based on the data in Table 2, it can be said that the research participants are mature, as they, including the males, whose number reached (172) individuals, are considered to be at the peak of their productivity in terms of age, since most of them fall within the middle age range, between (31-50 years), with some exceptions that reached (21.6%) of those who were older than this age range. On the other hand, the majority of the research participants held a bachelor’s degree, as their percentage constituted (51.4%). The results of this description indicate the ability of the study sample to answer the questions posed in the questionnaire by the researchers and the existence of an initial understanding of the research variables and dimensions.

**Table 2.** Description of the individuals surveyed.

Sex			
Female		male	
Number	%	Number	%
**172**	**54**	**147**	**46**

Certificate (scientific qualification)					
Higher degree		Bachelor's		Preparatory school and below	
Number	%	Number	%	Number	%
10.9	41	78.9	295	10.2	38

Age							
20-30		31-40		41-50		50 and more	
Number	%	Number	%	Number	%	Number	%
**51**	**16**	**112**	**35.1**	**87**	**27.3**	**69**	**21.6**

Source: By authors.

### 4.2 Description and diagnosis of the research variables

1. **Description and Diagnosis of the Digital Footprint Variable**: The data in Table 3 reveal that there is consensus among respondents regarding whether or not all questionnaire variables are measurements of the Digital Footprint. The accumulating consistency rate of the positively answered ones (strongly agree and agree) in general was 77.64% for all respondents. This shows a level of agreement in the answers given by respondents to the items that make up this variable and reflects that participants’ evaluations tend to be positive, as measured on a five-point Likert scale. This is also reinforced by the mean (4.005),which is greater than the assumed mean (3) and its standard deviation (0.892). This represents a unanimous uniformity among the responses to these variables, in line with the personal perceptions of all respondents.
2. **Description and Diagnosis of The Customer Experience Variable:** In Table 4, it is clear that there ere is a consensus between respondents about the opinion of items from the client variable with its data. The total agreement rate for respondents yes (strongly agree,agree) responses was 81.48%. This signals a large consensus of agreement of respondents on the items of this variable, which expresses that they lean towards being positive, as measured by the 5-point Likert scale. By a mean of 4.113 (above the expected value of 3) and a standard deviation of 0.852. The proportion of neutrals was 13.32%, a figure smaller than the agreed in percentage. This means that there is a joint consensus among the respondents regarding their understanding of these variables.

**Table 3.** Description and diagnosis of digital footprint variants.

Variable	Mean	S.D	Variable	Mean	S.D
x1	3.266	1.231	x9	4.633	0.604
x2	4.316	0.746	x10	4.451	0.569
x3	4.344	0.756	x11	4.457	0.642
x4	3.909	1.049	x12	4.363	0.638
x5	3.548	1.448	x13	3.984	0.852
x6	4.181	0.860	x14	4.250	0.756
x7	3.924	1.055	x15	4.065	0.853
x8	2.391	1.329	**General Average**	**4.005**	**0.892**

Source: By authors.

**Table 4.** Shows the definition and diagnosis of the customer experience variable.

Variable	Mean	S.D	Variable	Mean	S.D
y1	4.348	0.744	y11	4.106	0.761
y2	4.210	0.786	y12	4.163	0.807
y3	3.862	0.954	y13	3.924	1.031
y4	4.122	0.832	y14	4.197	0.736
y5	4.103	1.066	y15	4.141	0.774
y6	3.689	1.119	y16	4.137	0.804
y7	4.294	0.769	Y17	4.247	0.845
y8	4.197	0.765	Y18	4.084	0.915
y9	4.332	0.758	Y19	4.021	0.929
y10	3.771	0.890	Y20	4.329	0.757
**General Average**				**4.113**	**0.852**

Source: By authors N = 319.

### 4.3 Testing the research hypotheses

1. **Analyzing the correlation between digital footprint and customer experience**

**Figure 2.**
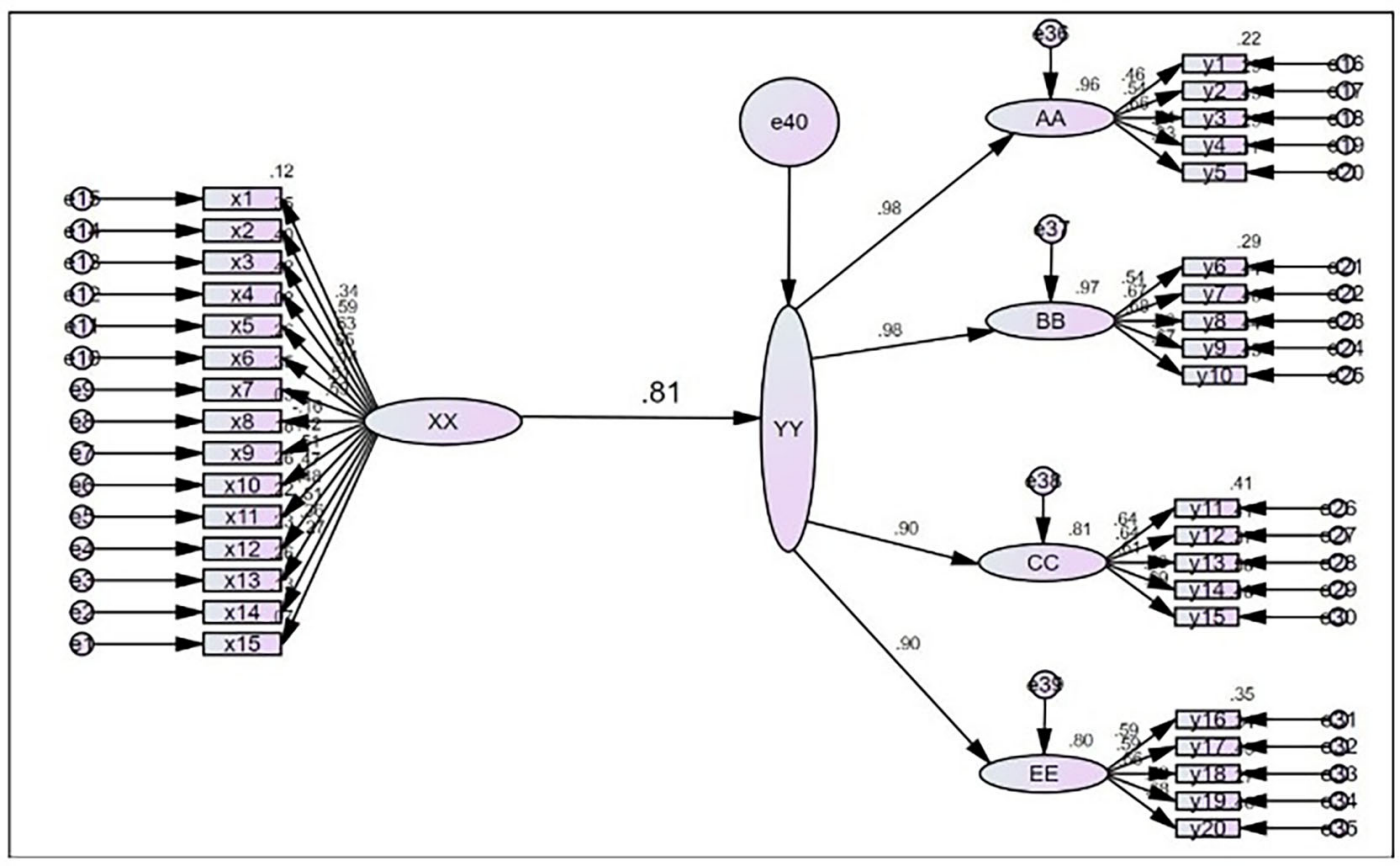
Illustrates the correlations between these two variables.

The results in Table 5 confirm that the two research variables are significantly correlated with each other, as in terms of their high correlation coefficient value estimated at (0.60) and level of significance designated by p-value equal to (0.000), which is much less than (0.05) ( Table 5). These findings suggest that Digital Footprinting improves customer experience when customers read or purchase products/services. Therefore, the first main hypothesis can be rejected, and the alternative hypothesis, which states that there is a correlation between digital footprinting and customer experience, can be accepted. The Figure 2 illustrates the correlations between these two variables.

2. **Analysis of the Effect Relationship between Digital Footprint and Customer Experience**

**Table 5.** The relationship between digital footprint and customer experience.

Correlations		Digital footprint
Customer experience	Pearson Correlation	0.60 ^**^
	P-Value	0.000
	N	319

Source: By authors.

**Figure 3.**
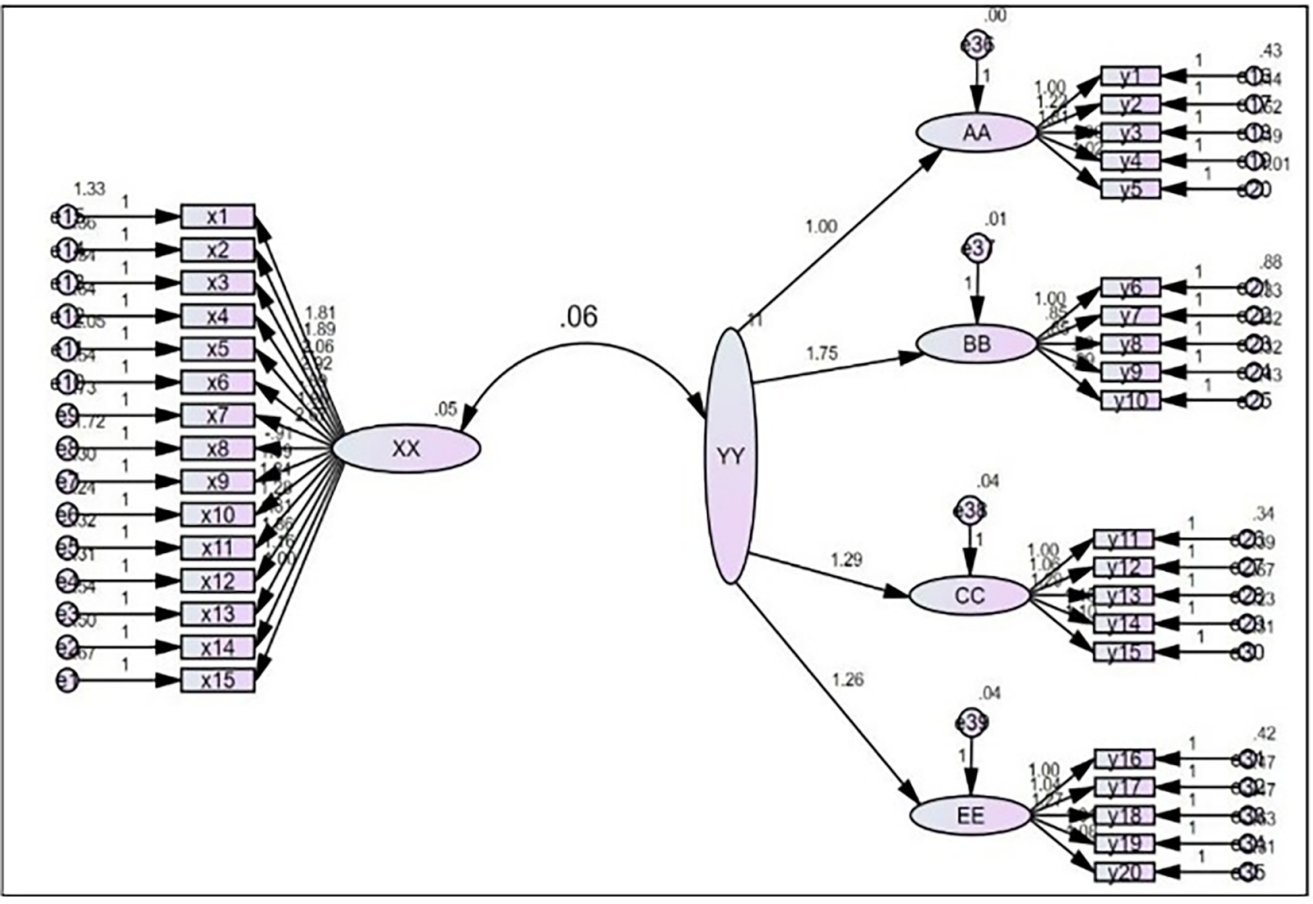
Shows the analysis of the direct effect of the digital footprint on customer experience.

This hypothesis states that the Digital Footprint has a statistically significant effect on customer experience. This effect is determined in Table 6 of the regression analysis results shows a significant effect of digital footprint as an independent variable on customer experience as a dependent variable. The calculated F-value (202.896) was greater than the critical F-value (3.84) at 1.308 degrees of freedom and a significance level of 0.05. The coefficient of determination (R ^2^) was 39%, indicating that the explained differences in customer experience were due to the Digital Footprint effect, whereas the remaining differences were attributed to random variables that could not be controlled or were not included in the regression model. Following up on the value of the β1 coefficient, which is (0.826), and performing a t-test, it was found that the calculated t-value was (14.244), which is a significant value and greater than its critical value (1.645) at a significance level of (0.05) and two degrees of freedom (1.308). Figure 3 shows the analysis of the direct effect of the digital footprint on customer experience.

**Table 6.** Analysis of the effect relationship between digital footprint and customer experience.

Independent variable	Direction of the relationship	Dependent variable	Estimate	Std. error	Confidence interval 95%		R ^2^	F	P-value
Digital Footprint	→	Customer experience	0.806	0.233	0.347	1.265	39	202.896	0.000
			0.826	0.058	0.712	0.940			

Source: Prepared by the researchers based on the results of the statistical program SPSS.

## 5. Results discussion

The research results shows that the Internet service companies in Nineveh Governorate, use a digital footprint data as tool to gather customers’ information from various digital platforms, but there is unsophistication from the analytical point of view for processing such data and up-to-date it is not used effectively to enhance and improve customer experience. This observation echoes the findings of ( Koidl et al., 2018) who emphasized that many firms store silos full of digital customer data with little possibility of translating this data into value and, as such, are inefficient and ineffective in using it.

The survey also found that a better customer experience can be achieved if organizations use digital interaction data to personalize the service provided to their customers, driving increased satisfaction and loyalty between the individual and the organization.

In addition, this study showed that there is poor long-term corporate memory in the awareness of the strategic value of customer digital trails. This finding is in agreement with the observation of ( Martin & Murphy, 2016) pointing out that a variety of limitations pose barriers for organizations to realize full advantage from digital footprint data.

Furthermore, the study explained that the surveyed organizations do not give much importance to sophisticated data analytical tools. This aligns with the views of ( Pine & Gilmore, 2021) which argues that evolving from the “service economy” to the “experience economy” requires not only availability of the actual customer data, but also a set of analytics capabilities on how organizations may turn insight from customer data into enriching and value adding experiences.

However, the study found clear awareness among the respondents regarding the two research variables, as observed through the descriptive and diagnostic results generated from the data after analysis. This was in addition to confirming the presence of both an effect relationship and a correlation relationship between the studied variables.

### 5.1 Conclusion

Our study confirms the recent trends in organizations that support data analytics and support the digital footprint as a strategic resource that contributes to improving customer experience. The study also reveals a gap in on-premises applications in developing environments as a result of the poor use of data analytics to improve loyalty and personalize actual customer experiences. This will allow for future theoretical and practical research at the corporate level, especially in the service sector.

### 5.2 Limitations and future research

The current study was limited to a sample of Internet service providers in Nineveh Governorate, which limits the possibility of generalizing the results to other service sectors with different geographical contexts. It should also be noted that the study relied on data collected from questionnaires, and this would be affected by the different levels of respondents’ digital maturity. In addition, the study focused on the relationship between digital prints and customer experience without delving into the intermediate, modified, or control variables that may affect the nature of the relationship. Therefore, this study proposes to direct future research towards studying the role of advanced analytics and artificial intelligence in enhancing the understanding of customer behavior based on their digital footprint, as well as expanding the study to include other service sectors or compare companies with different levels of digital maturity.

## Ethical approval

Our institution when we conducted the study (the University of Mosul, Iraq), did not have an Institutional Review Board (IRB) or official ethics committee specifically assigned to review social science research. For this reason, it was not feasible to gain formal ethical approval. However, we strictly adhered to internationally accepted ethical standards for studies including human subjects. Portions of participation was voluntary, obtained informed consent electronically at the first page of the questionnaire, there were no collected personal identification data and confidentiality/anonymity full respect throughout the study.

## Data Availability

-
**Primary Data:** The primary data supporting the findings of this study were collected using a structured survey questionnaire and analyzed using SPSS software. The dataset includes respondents’ demographic information (gender, age, and educational level) as well as responses to the study variables measured on a five-point Likert scale. Due to ethical considerations and data protection requirements, the raw dataset (SPSS file) is not publicly available but is available from the corresponding author upon reasonable request and in accordance with ethical guidelines. -
**Extended Data:** The extended data consist of the survey questionnaire used for data collection. This file has been publicly deposited in the Zenodo repository under a Creative Commons CC0 1.0 Universal dedication and can be accessed at the following DOI: https://doi.org/10.5281/zenodo.17989147 ( Al-Jubory et al., 2025) The corresponding author may provide additional materials upon reasonable request and in compliance with ethical and data protection principles. Corresponding author email: mohamed_almola@uomosul.edu.iq
