# Digital Footprint and Its Impact on Improving Customer Experience: An Exploratory Study of a Sample of Internet Companies in Nineveh Governorate

**DOI:** 10.12688/f1000research.174437.2

**Published:** 2026-04-30

**Authors:** Abdul Sattar Salim Awadh Al – Jubory, Saif Khalid Zakaria, Mohammad Mahmood Al-Mulla Hasan

**Affiliations:** 1 Business Administration, University of Kirkuk, Kirkuk, Kirkuk Governorate, Iraq; 2 Management Information Systems, University of Mosul, Mosul, Nineveh Governorate, Iraq; 3 Marketing Management, University of Mosul, Mosul, Nineveh Governorate, Iraq

**Keywords:** Digital Footprint, Customer Experience, Personalization, Internet Service Providers, Nineveh.

## Abstract

**Background:**

Despite Internet Service Providers (ISPs) collects and builds customers’ digital traces, very limited evidence exists in the contexts of Iraqi (ISPs) on how such data are utilized for customer experience (CX) enhancements.

**Methodology:**

Data were collected through online structured questionnaire from 319 employees and managers of different (ISPs) in Nineveh Governorate/Iraq. The study used the individual respondent as the unit of analysis and evaluated customer-experience enhancement from the viewpoint of (ISPs). Digital footprint practices and the perceived improvement of (CX) were assessed using a five-point Likert scale. Descriptive statistics, reliability analysis, correlation tests & simple linear regression were used to analyze the data.

**Results:**

However, the results of statistical analysis show that digital footprints can significantly drive service personalization and operational efficiency. However, the results indicate a strong implementation gap between data collection and its fulfillment for enabling customer interactions creating value.

**Conclusion:**

This paper concludes with a salient insight; for (ISPs) to use the digital Footprint of their customers and residual data left by these customers towards improving the quality of their offerings, they should embrace an approach that is balanced, evidence-based alongside transparent accounting for privacy concerns that underpins user trust.

## Introduction

As the pace of technological change accelerates, firms are being urged to develop — and ultimately manage customer experience (CX) more fully within increasingly blurred markets (and offering), greater organizational agility, and customer centricity ( Lemon & Verhoef, 2016). Consequently, (CX) has emerged as a major source of competitive differentiation. This transformation is particularly evident in internet-based services, where customers increasingly interact with Internet Service Providers (ISPs) through digital channels and smart devices. Within this context, such interactions generate digital footprints that provide firms with valuable behavioral insights; Accordingly, digital footprints have acquired growing strategic importance because they enable firms to personalize their offerings, improve service delivery processes, and enhance (CX) outcomes ( Hassan, 2024).

From the foregoing, the concept of digital footprints has evolved: the trace of user data when users are online. It is dynamic and constantly updated, and is a medium for predicting behavior or preference, making it useful for business ( Neelam et al., 2025). ( Koidl et al., 2018) found that it is possible to systematically retrieve and analyze digital traces, so companies know their customers in detail, predict hidden needs for them, and make personalized offers and proactive support available. The digitization of information and data capture has become an increasingly appealing concern for companies seeking to foster a better (CX), although it is far from free of ethical and strategic conflict ( Wedel & Kannan, 2016). On the other hand, opaque data practices could challenge faith, pose privacy risks, and make customers feel that they are being watched too much, which needs a balance ( Martin & Murphy, 2016). Furthermore, the potential of digital footprints varies depending on several parameters, such as transparent governance, responsible data treatment, and customer awareness of the collection and usage of their data ( Acquisti et al., 2015). However, despite the increasing content on digital personalization and data related to consumers ( Wedel & Kannan, 2016), companies should train existing employees in data analysis and hire new staff with such capabilities because it is crucial in the near future. However, research on how digital footprints specifically contribute to improving (CX) in Internet service markets is still scarce, while comparing this process between developing contexts and mature ones, where the services, digital culture, and customers’ minds vary, is contextual.

A preliminary structured search in the major academic databases, including Scopus and Web of Science, with keywords like “digital footprints,” “(CX)” and (ISPs) shows that the existing studies are typically focused on developed economies yet mostly address generic-data analytics or big data applications. In contrast, limited attention has been given to the role of digital footprints in shaping (CX) within Internet service contexts in developing regions, particularly in Iraq. This indicates a clear contextual and conceptual gap in the current body of knowledge.

Based on this, the aim of this study is to explore the role of digital footprints in improving (CX) within Internet service companies in Nineveh Governorate, Iraq, through

1. To analyze the extent to which (ISPs) utilize customers’ digital footprint data within their current operational and service practices.
2. To identify the level of deployment of digital footprint data in enhancing (CX) among (ISPs).
3. To examine the relationship and impact of digital footprints on improving (CX) within (ISPs).

## 1. Literature review

A literature review is also important for situating the current study from previous research studies and describing the research gap more precisely. To this effect, ( Dhal et al., 2025) pointed out that technological progress has also had a positive impact on (CX) enhancement. The ubiquitous use and evolution of digital platforms have allowed businesses to accumulate and analyze digital footprints to enhance (CX), particularly in the flourishing service industry ( Arya et al., 2019).

On the other hand, ( Micheli et al., 2018) highlighted that these digital footprints represent one of the dimensions of digital inequality, considering all the data left by users that can be exploited to customize services and hence improve user experience. Some people own their data, and some do not, and companies that use these digital trails in the right way will make enormous economic and marketing gains.

Conversely, the research by ( Martin & Murphy, 2016) uncovered a series of key issues around privacy perceptions, data transparency, and trust in relation to the use of customers’ digital foot research, showing that personalized services improve the quality of experience, yet too much or non-transparent data tracking can result in concerns, thus passively affecting customers’ attitudes. Moreover, digital trace advantages are not uniformly available among social groups and individuals as there is variation in digital culture. Different levels of internet use may affect the degree to which various relationships can be improved or experience difficulties ( Micheli et al., 2018).

We conclude from the above that there is a growing interest in the concept of digital footprint, but empirical evidence in developing contexts is limited, particularly regarding how (ISPs) use digital footprints to improve (CX) in areas such as Nineveh Governorate, as the level of digital adoption and customer awareness varies from region to region. To fill this gap, this study aims at exploring how ISPs in Nineveh avail the digital footprint data to improve (CX).

### 1.1 Study problem

Research shows that digital Footprint can have a double-edged sword effect on (CX), in it is provided digitally. These technologies make it possible to offer a more personalized service and tailor services and products according to preferences, which boosts customer satisfaction and engagement. However, this type of data collection can also evoke privacy and trust concerns with customers and might hinder customers’ full adoption of digital features ( Martin & Murphy, 2016). This requires a balance in their study. Additionally, the studies proved that digital footprint open new research opportunities for understanding (CX). But, they also pose methodological challenges because they are dynamic over time and challenging to generalize findings outside of the analysis sample ( Golder & Macy, 2014). Empirical evidence on the effects of digital Footprint is scarce in the context of (ISPs), and what exists has found inconclusive results indicating a clear research gap that leads to exploring how digital Footprint impact (CX).

Background No studies were found that examined how ISPs function in Nineveh Governorate (Iraq) particularly regarding the exploitation of digital customer traces and their role in shaping/delivering and personalizing the customer journey or how those data-led strategies are being perceived [i.e., cognitive and behavioral responses]. Thus, there are contextual studies on and regarding mechanisms of using digital footprints for (CX) improvement in this local service context along with their strategic deployment. Hence, the research problem could be simplified as follow:

• How Digital Footprint impact internet users and how to utilize this data for better service personalization and improved (CX) from the (ISPs) side in Iraq’s digital transformation context?

### 1.2 The importance of the study

This research deals with the relevance of said attributes, which leads to:

1. The importance of this study stems from the clear expansion of the digital environment in Iraq, where the number of internet users reached 38.0 million in January 2025, representing 81.7% of the total population. This reflects the growing scale of digital interactions and the increasing volume of digital footprints generated through online activities ( Kemp, 2025).
2. Its importance also derives from the fact that institutional assessments in Iraq indicate that digital maturity remains at a basic level across 14 areas of e-governance, highlighting the need to strengthen the organizational and technical capacities required for the effective management and utilization of digital data ( UNDP, 2024).
3. This issue becomes even more important in the Internet service provider sector, as it is one of the sectors most directly connected to users’ digital interactions. Accordingly, examining this sector in a local context such as Nineveh can provide useful insights into how digital footprints may be leveraged to improve (CX) in a developing setting such as Iraq ( Kemp, 2025; UNDP, 2023).

### 1.3 Study objectives

1. Identify best practices for employing a customer’s digital footprint to enhance the (CX).
2. Assess the impact of using a customer’s digital footprint on improving (CX)s.
3. Identify evidence-based strategies for the effective and ethical use of digital footprints within local ISP contexts.

## 2. Conceptual framework

### 2.1 Digital footprint

The concept of *digital footprint* first emerged under the expression “the snail trail,” as mentioned by ( Negroponte, 2015), and was later termed “data remnants” by Tim O’Reilly. Initially, it referred only to traces of information left behind after browsing the internet. Today, however, the term digital footprint or digital shadow refers to the data generated and used, regardless of the type of device involved ( Kligienė, 2012).

According to ( Majeed et al., 2020), a Digital Footprint can be described as a digital file or shadow that constantly follows the user, such as a tattoo that cannot be easily erased as it continues to exist even after a person tries to remove it. ( Kligienė, 2012) further explains that the digital footprint represents the impact of an individual’s actions in the digital environment, including the use of mobile phones, television, the internet, or any other device equipped with sensors. This term is relevant not only to persons but also to organizations, as it describes a broad spectrum of online actions, communication, and behavior ( Golder & Macy, 2014). In line with ( Arya et al., 2019), digital footprints are the collection of data or information about an individual that is intentionally and unintentionally left behind, while being active on the Internet, such as visiting websites or using online social networking sites. The desire to learn customer behavior in a digital economy and in a digital environment rather than in a traditional market leads to a substantial increase in interest in the study of digital footprint because Internet use is increasing ( Rauniar et al., 2013). In addition, the expansion of mobile industries, 4G networks, and cloud computing has boosted the adoption of social media, which has led to greater adoption by consumers on smart devices and thus an increase in the size of digital footprints. Users constantly create digital footprints through comments, photos, videos, blogs, ratings, e-shopping, and interactions with the government and service apps. Studies conducted by ( Charlesworth, 2014) show that digital footprints reveal users’ interests, social identities, cultural backgrounds, professional affiliations, and geographical connections, making them particularly valuable for companies wishing to track customer behavior and build personal profiles for them. Thus, the digital footprint can be understood as user-related information within the digital environment.

### 2.2 Types of customer digital footprints

The literature and research indicate that customer Digital Footprints fall into two main categories: active and passive Digital Footprints.

1. Active Digital Footprints: These types of data refer to data that users intentionally create and share during their online interactions ( McDermot, 2018).
2. On the other hand, Passive Digital Footprints are collected without user express knowledge through background data gathering inherent in websites, apps and network infrastructures ( Micheli et al., 2018). Such passive digital footprints include background-related data like browsing behavior, click patterns, device-generated information, and location traces obtained through online interactions. Such data are often leveraged in digital marketing contexts to estimate consumer profiles and aid personalization and ad decisions ( Estrada-Jiménez et al., 2017). According to recent research, there is a growing dependence of platforms on passive behavioral data, which not only personalizes digital experiences but also manipulates decision-making processes.

Consequently, the difference between these two kinds of data has become a key area of consideration regarding privacy, data ethics, and customer autonomy within the digital marketplace ( Zuboff, 2019).

As a result, the distinction between these two types of data has become central to discussions on privacy, data ethics, and customer autonomy in the digital marketplace ( Zuboff, 2019).

### 2.3 Advantages and disadvantages of customer digital footprints

Table 1 outlines the advantages and disadvantages of a customer’s Digital Footprint.

**Table 1.** Advantages and disadvantages of customer digital footprints.

Aspect	Advantages (Positive implications)	Disadvantages (Passive implications)	Source
**Personalization**	Enables companies to personalize content, recommendations, and marketing messages based on customer interests, improving relevance and engagement.	Excessive personalization may lead to a sense of surveillance and loss of autonomy, passively affecting trust.	( Robles-Carrillo, 2024)
**User Experience**	Enhances customer experience by providing smoother navigation, tailored service offerings, and predictive support features.	The complexity and volume of digital trace data make it difficult for users to manage or control their digital identity over time.	( Hassan, 2024)
**Social Interaction**	Improves social connectivity by suggesting relevant communities, friends, and content based on shared interests across social networks.	Customer profiling can be used to infer sensitive personal attributes, raising ethical concerns about data misuse.	( Kligienė, 2012)
**Location-Based Services**	Enables accurate and efficient delivery of location-based services such as mapping, local recommendations, and service coverage optimization.	Passive and continuous collection of geolocation data may occur without the user’s explicit awareness or consent.	( Arya et al., 2019)

Source: By authors.

### 2.4 The concept of customer experience

Researchers define (CX) as the accumulated impressions of the customer across all points of direct and indirect interaction with the company and cover the pre-, during-, and post-consumption stages; that is, it is not a single event, but rather an interactive journey that includes perception, emotion, and cognitive evaluation as a result of the customer’s interaction with the brand through multiple channels ( Lemon & Verhoef, 2016). However ( Gahler et al., 2023) highlighted that (CX) is shaped by the functional, emotional, and social aspects of interaction that make it a personal response that varies from client to client based on individual expectations. while ( Hardcastle et al., 2025) explained that companies are increasingly focusing on designing integrated experiences across digital channels, which requires understanding customer needs, analyzing their data, and personalizing interactions to enhance the perceived value of the relationship. However ( Homburg et al., 2020) argued that (CX) is closely related to the level of customer engagement in interacting with the service provider, making it a collaborative process rather than a one-way process. We believe that it is a multidimensional response and is determined by the extent to which the organization manages its touchpoints and valuable experiences across the various digital channels of services.

### 2.5 Customer experience dimensions

(CX) is a dual-direction, multi-layered process that scholars describe through several interconnected dimensions.

1. **Relational Dimension**: It relates to the quality of long-term relationships that customers and companies have with each other, which comprises elements such as trust, customization, and clear communication. All these factors are paramount for value-in-use and loyalty ( Ponsignon et al., 2017).
2. **Service, Physical environment**: Factors in this dimension are the usability and accessibility of service places and making digital and physical service touchpoints user-friendly. These determinants in combination affect customers’ cognitive appraisals and emotions during service encounters ( Dong et al., 2008).
3. **The social layer of the experience** of the behavior and action of other users is created in this layer when others influence and create a sense of belonging and how they can shape the emotional aspect too ( Siqueira Junior et al., 2025).
4. **Organizational Ability to Deliver Dependable and Responsive Service**: This relates to the firm’s customer service as well as the speed and accuracy of a company’s response to effectively solve problems that enhance satisfaction and increase repurchase ( Santos-Vijande et al., 2012).

In light of these perspectives, and based on the literature concerning digital footprints and (CX), 7th conceptual model of the current study is based on the assumption that digital Footprint generate behavioral information that can help companies better understand customer interactions, support personalization, and improve service delivery processes. Since the (CX) is shaped across multiple touchpoints throughout the customer journey, the effective use of this data is expected to contribute to a perceived improvement in the (CX). Therefore, the model assumes a direct positive relationship between the use of digital Footprint and the perceived improvement in the (CX).

**Figure 1.**
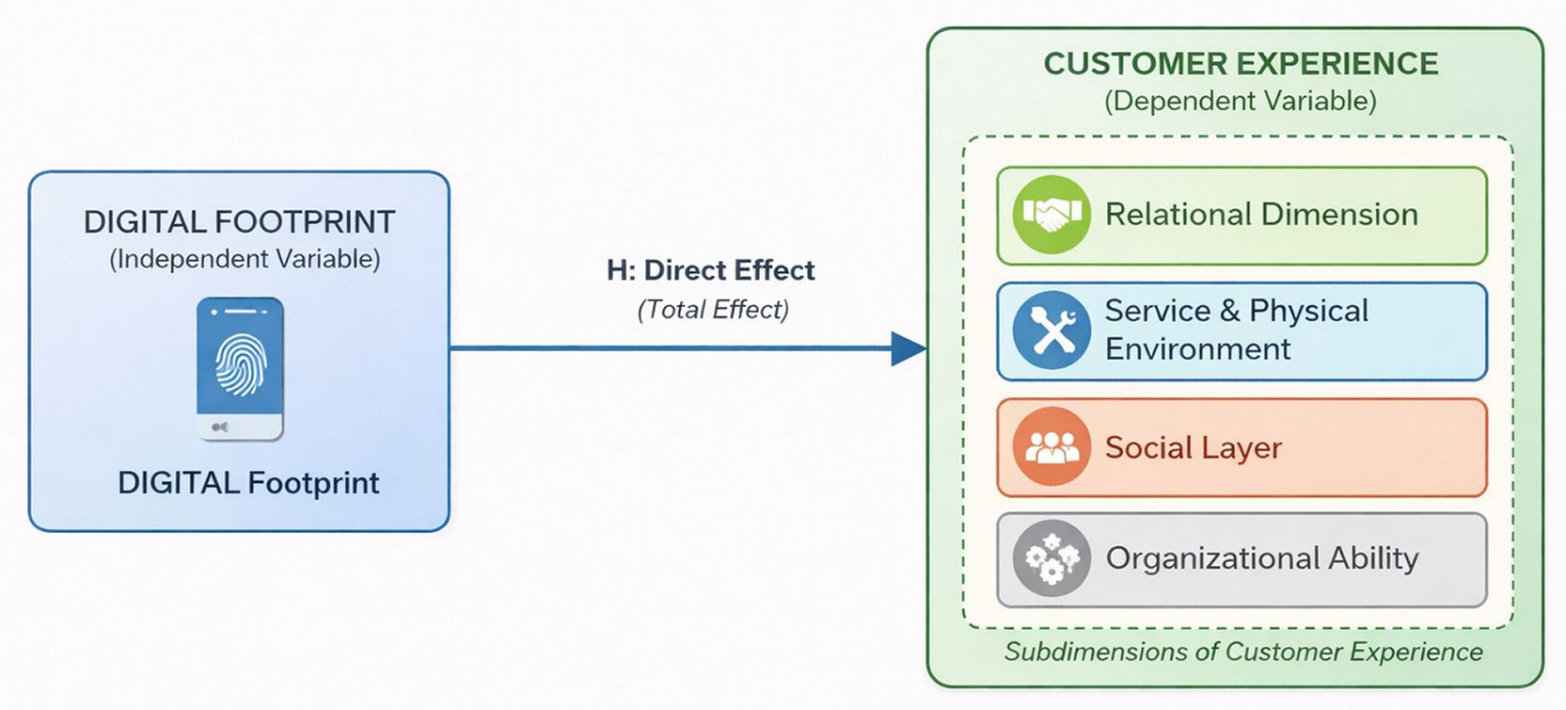
Conceptual model of the study. This figure presents the proposed conceptual model illustrating the relationship between digital footprints and (CX) in Internet service companies.

Based on the proposed model in Figure 1 and the rationale previously explained, we formulated the following hypotheses:

• Digital footprint use is positively associated with perceived (CX) improvement in the surveyed ISPs.
• Digital footprint use has a positive and statistically significant effect on perceived (CX) improvement in the surveyed ISPs.

## 3. Methods

**Research Population and Sample:** Nour Al-Badia was identified as the principal internet service provider in Nineveh Governorate, with 58 subsidiary service providers operating under its network. Based on Directorate of Transport and Communications statistics ( Central Statistical Organization, 2025), the total working individuals from these companies were 5,755 which formed the base population of this study. A link for an electronic questionnaire was sent directly to a sample of 390 people, and 319 responses were collected for analysis purposes. The discrepancy between the number of distributed and returned questionnaires could be explained by viewing that some potential participants had not enough time or refused to be in study. In order to handle ethical issues, the authors implemented an informed consent process on the first page of the electronic form. Participants were explicitly made aware that the study was being conducted, their participation was voluntary, and they could withdraw at any time without implications. They were also told that the questionnaire would not collect personally identifiable or sensitive information, and that all responses would be kept strictly confidential.

**Questionnaire Form**: The questionnaire is the principal tool used in this study for collecting data regarding research variables. It was made up of 35 items distributed between two variables:

• Customer Digital Footprint: Measured with 15 items.
• (CX): Measured with 20 items.

A five-point Likert scale was used to evaluate each item (1 = Strongly Disagree, 5 = Strongly Agree).

Default Items were adapted from existing research tools, while others were developed to fit the local context of Mosul. The validity of the content was tested by a number of digital marketing experts, who helped to review the questionnaire and verify the clarity of each item and their appropriation with respect to the research objective.

Cronbach’s alpha was used to test the reliability of this questionnaire. The results indicated that the participants’ digital footprint of their responses, with an average of 0.661 and specific item scores from 0.615 to 0.724, reported as Cronbach’s alpha ≥0.7 denoting accepted internal consistency between instrument elements. In contrast, overall (CX) was scoring very high (with a score of 0.905), meaning that the perceived quality of the experience is bigger than its Quantity in Terms of Digital Interaction. (CX) is not just about where you are or how much, but the interaction itself. No convergent or discriminant validation assessments were conducted and no confirmatory factor analysis was performed. But the researcher thinks these steps are a pivotal point in elaboration of future instruments and in bolstering the purported structure of the dimensions. These initial findings are solid indicators of the relationship between digital footprint and (CX), demonstrating that visibility is a means to an end (enhancing experience), not an end in itself.

The one-way Harman test was also administered to identify the effect of cross-style bias between items representing independent variable (digital footprint) and dependent variable (CX). With only 28.3% of the total variance accounted for by the first factor, well below what is considered a threshold (50%) for significant bias. Which indicates that the data do not manifest the predominance of a single factor across all items and that the items measure several dimensions, as expected: making it less likely that there will be cross-style bias in this study.

**Statistical Methods Used:** Questionnaires were utilized to gather data on information such as frequencies, percentages, means, and standard deviations. Then, using Pearson’s correlation coefficient to evaluate the strength of relationship between variables and simple linear regression model to measure the impact of independent variables on dependent variable.

## 4. Description and diagnosis of the research variables and testing of its hypotheses

**This section will include three paragraphs:**

### 4.1 Description of the research participants

According to Table 2, the bulk of research subjects are in the middle-aged category (31–50 years), and consists of 172 males, while only a few percentage 21.6% is surpassed this list. Considering academic achievement, most subjects have a bachelor degree (51.4%). The distribution indicates the ability of respondents to comprehend and analyze study questionnaire while demonstrating relevant representation across age categories and educational levels.

**Table 2.** Description of the individuals surveyed.

Sex			
Female		male	
Number	%	Number	%
**172**	**54**	**147**	**46**

Certificate (scientific qualification)					
Higher degree		Bachelor’s		Preparatory school and below	
Number	%	Number	%	Number	%
10.9	41	78.9	295	10.2	38

Age							
20-30		31-40		41-50		50 and more	
Number	%	Number	%	Number	%	Number	%
**51**	**16**	**112**	**35.1**	**87**	**27.3**	**69**	**21.6**

Source: By authors.

### 4.2 Description and diagnosis of the research variables

1. **Description and Diagnosis of the Digital Footprint Variable:** The data in Table 3 reveal that there is consensus among respondents regarding whether or not all questionnaire variables are measurements of the Digital Footprint. The accumulating consistency rate of the positively answered ones (strongly agree and agree) in general was 77.64% for all respondents. This shows a level of agreement in the answers given by respondents to the items that make up this variable and reflects that participants’ evaluations tend to be positive, as measured on a five-point Likert scale. This is also reinforced by the mean (4.005),which is greater than the assumed mean (3) and its standard deviation (0.892). This represents a unanimous uniformity among the responses to these variables, in line with the personal perceptions of all respondents.
2. **Description and Diagnosis of The (CX) Variable:** In Table 4, it is clear that there ere is a consensus between respondents about the opinion of items from the client variable with its data. The total agreement rate for respondents yes (strongly agree, agree) responses was 81.48%. This signals a large consensus of agreement of respondents on the items of this variable, which expresses that they lean towards being positive, as measured by the 5-point Likert scale. By a mean of 4.113 (above the expected value of 3) and a standard deviation of 0.852. The proportion of neutrals was 13.32%, a figure smaller than the agreed in percentage. This means that there is a joint consensus among the respondents regarding their understanding of these variables.

**Table 3.** Description and diagnosis of digital footprint variants.

Variable	Mean	S.D	Variable	Mean	S.D
The company relies on diverse digital platforms to enhance its online presence.	3.266	1.231	The company protects customer data through effective digital security systems.	4.633	0.604
The company has a regularly updated and easily accessible website.	4.316	0.746	The company's digital interface (app, website) is user-friendly and intuitive.	4.451	0.569
The company provides accurate information about its services through online channels.	4.344	0.756	The company uses digital advertising to connect with prospective customers.	4.457	0.642
The company responds quickly to comments posted on its digital pages.	3.909	1.049	Digital content — constantly evolving to lure customers.	4.363	0.638
The company regularly interacts with customers via social media.	3.548	1.448	About company clear information on customer privacy and digital data.	3.984	0.852
The company publishes regularly updated digital content about its online services.	4.181	0.860	Digital analytics technology is employed to gain insight on consumer behavior.	4.250	0.756
The company utilizes online customer behavior tracking technologies to improve its services.	3.924	1.055	A brand is built with a digital presence that focuses on the current state of the company.	4.065	0.853
The company provides reliable digital communication channels to resolve customer issues.	2.391	1.329	**General Average**	**4.005**	**0.892**

**Table 4.** Description and diagnosis of the customer experience variable.

Variable	Mean	S.D	Variable	Mean	S.D
I find the internet service subscription process clear and easy.	4.348	0.744	I feel the company genuinely values ​​customer feedback.	4.106	0.761
I am provided with all the information I need before subscribing.	4.210	0.786	The maintenance and technical support procedures are clear and prompt.	4.163	0.807
The quality of the internet service meets my expectations.	3.862	0.954	I feel confident dealing with the company.	3.924	1.031
The internet service is stable and does not experience frequent outages.	4.122	0.832	The company's app or website is user-friendly.	4.197	0.736
The internet speed provided matches what was agreed upon.	4.103	1.066	The information the company provides online is reliable and accurate.	4.141	0.774
The customer service staff treat me with respect and professionalism.	3.689	1.119	My experience with the company has led me to continue using their services.	4.137	0.804
My issues are resolved quickly when I contact customer service.	4.294	0.769	The additional services offered by the company enhance my experience.	4.247	0.845
The company provides convenient communication channels (phone, app, chat, etc.)..	4.197	0.765	I am generally satisfied with the internet service provided.	4.084	0.915
Based on my experience, I recommend the company to others	4.332	0.758	The company's service meets my expectations before subscribing.	4.021	0.929
The service pricing is reasonable considering its quality.	3.771	0.890	My positive experience makes me less inclined to switch to another company.	4.329	0.757
**General Average**				**4.113**	**0.852**

### 4.3 Testing the research hypotheses

1. **Analyzing the correlation between digital footprint and customer experience**

The results in Table 5 confirm that the two research variables are significantly correlated with each other, as in terms of their high correlation coefficient value estimated at (0.60) and level of significance designated by p-value equal to (0.000), which is much less than (0.05) ( Table 5). These findings suggest that Digital Footprinting improves (CX) when customers read or purchase products/services. Therefore, the first main hypothesis can be rejected, and the alternative hypothesis, which states that there is a correlation between digital footprinting and (CX), can be accepted.

2. **Analysis of the Effect Relationship between Digital Footprint and (CX)**

**Table 5.** The relationship between digital footprint and customer experience.

Correlations		Digital footprint
Customer experience	Pearson Correlation	0.60 ^**^
	P-Value	0.000
	N	319

Source: By authors.

**Figure 2.**
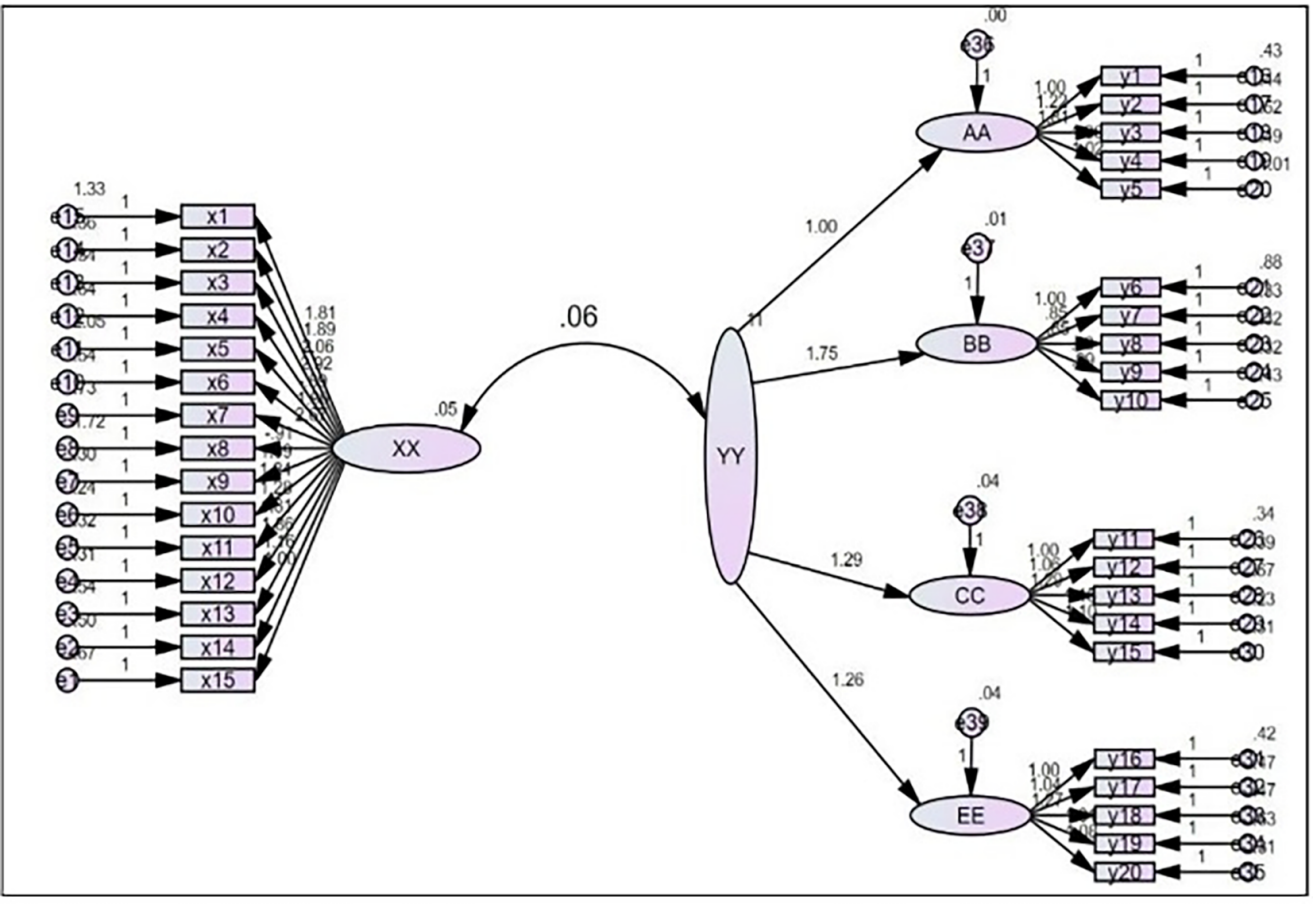
Demonstrates the analysis of direct digital footprint impact on customer experience.

This hypothesis states that the Digital Footprint has a statistically significant effect on (CX). This effect is determined in Table 6, where the effect of digital footprint as the independent variable on (CX) as the dependent variable reached a calculated F-value of 202.896, which is greater than the critical F-value of 3.84 at 1.308 degrees of freedom and a significance level of 0.05. The β _1_ coefficient was 0.826, with a calculated t-value of 14.244, which is greater than the critical value of 1.645, indicating a strong statistical significance for the relationship. The coefficient of determination (R ^2^) of 0.39 suggests that digital footprint explains approximately 39% of the variance in (CX), while the remaining 61% reflects the presence of multiple other factors that may influence (CX), such as personal, environmental, social, and organizational variables, which were not measured within the scope of this research. From a methodological perspective, this unexplained variance highlights the limitations of a model relying on a single independent variable and underscores that (CX) is the product of a complex interaction between the digital footprint and multiple other factors. It indeed makes the interpretation of the results more complex as it is now about experience and tangible measurement. The direct effect of digital footprint on (CX) is depicted in Figure 2, which shows that the present results are well suited as reliable indicators for a first illustrative model due to the explained variance of current functioning, where residual variances linger from explaining variance (not all variation can be captured by linear models); in this case data indications will always display some unexplained values.

**Table 6.** The effect relationship analysis between the digital footprint and the customer experience.

Independent variable	Direction of the relationship	Dependent variable	Estimate	Std. error	Confidence interval 95%		R ^2^	F	P-value
Digital Footprint	→	Customer experience	0.806	0.233	0.347	1.265	39	202.896	0.000
			0.826	0.058	0.712	0.940			

Source: Prepared by the researchers based on the results of the statistical program SPSS.

We present the results of analyzing the direct effect from the digital footprint on (CX) in Figure 2.

## 5. Results discussion

This paper is organized around five lines of thought that are mutually interweaving: discussion about the key findings, how these relate to previous studies, the unique context surrounding Nineveh/Iraq, theoretical implications stemming from these results and their practical relevance for (ISPs).

Our results show a statistically significant positive correlation between the use of digital foot printing and improved (CX) in each of the ISPs considered. This finding indicates that the shuffled-off video data from user interactions might be a useful resource for gaining insight into the needs and preferences of customers, thus improving (CX) as seen by ISP employees. At the same time, the results highlight a clear shortfall between having digital data at your disposal and being able to use it analytically to drive more impactful improvements in (CX). This means that data acquisition merely does not imply organizational value unless such activities encompass analytical and organizational senses that can process data into useful knowledge.

This finding is reasonably congruent with the literature in confirming that digital data and digital footprints have increasingly emerged as an essential source of information for better insight into service usage, customer behavior patterns, preferences, needs and wants ( Lemon & Verhoef, 2016; Wedel & Kannan, 2016). facilitating the personalization of services and improvements in their delivery to enhance (CX). Additionally, it resonates with the assertion that digital footprints are not valuable to organizations themselves when merely collected; their worth lies in being analyzed and utilized toward developing better contextualized decisions and practices ( Micheli et al., 2018). In parallel, the findings of this paper confirm ( Koidl et al., 2018) critical insight into a disconnection between availability of digital data and actual capability to analyze such data in order to innovate (CX). However, the findings from the present study do not indicate a high degree of analytical maturity in companies studied. Rather, they expose a degree of utilization that is partial and incomplete. This resonates with the literature that discusses privacy and trust issues when working with digital data as a barrier to full use ( Martin & Murphy, 2016). Thus, whereas the study confirms the extant literature finding with respect to digital footprints strategic importance, it further illustrates how such substantiality does not necessarily lead to more advanced services unless conditioned by needed analytical and organizational capabilities at studied settings.

The relevance of this finding is more pronounced relative to Iraq specifically due the concomitantly expanding usage of ICT and electronic processes in the Iraqi digital environment over time, alongside a persistent demand to improve digital maturity as well as both institutional and organizational faculties around data ( Kemp, 2025; UNDP, 2023). The gap, as revealed in the study between data collection and its analytical utility enhance does not indicate a repetition of international literature but rather reflects features unique to this local context. These features include differences in digital infrastructure, inconsistent levels of institutional preparedness, low investment in specialized analytical capabilities and the relatively late embracement of data by some local service sectors. The internet service provider sector in Nineveh Governorate, among others, is of particular importance in this context as it represents one of the sectors most closely linked to users’ daily digital interactions. It is a strong proving ground for how digital footprints can become a concrete value with services.

Theoretically, these results add to the body of evidence supporting the proposition that digital footprints are more than just residuals of individuals’ use of technical systems but can be considered as a type of informational resource with potential if operated in an organizational context able to harness them in order to influence (CX). The findings further suggest that the link between digital footprint and (CX) needs to be approached not as a direct or mechanical relationship but rather by taking account of the organizational context, such as analytical capabilities, technological infrastructure readiness and organizational awareness of whether it is understood that turning data into service practices is useful. In this regard, the research makes an interpretative contribution to the body of literature by demonstrating that without having adequate organizational and analytical capabilities to complete, the cycle of digital footprint on (CX) may continue to remain narrow or incomplete.

As a practical consideration, the findings suggest that there is little need for (ISPs) to do any more than collect digital data and optimally analyze it and use it to improve service. Including the ingestion and analytical ways to log data within companies, design frameworks for keeping that information and enhance the connection between digital reads with decisions about who is served and when they also stress the need for a balanced approach to digital data, where maximizing the marketing benefits is only part of the equation — privacy and trust dimensions are important to take into account given how these aspects can affect the sustainability of the customer relationship. So, the study offers useful evidence that neither merely gathering data qualifies digital footprints as a competitive smoking gun in the internet services sector nor its real impact is at the service of actionable knowledge generated under self-determined and conscientious regulatory regimes.

### 5.1 Conclusion

Footprints in the digital world are an information component with strategic significance and valuable insight into (CX), which will be enhanced if such data through effective practices of understanding (the customer think), analyzing and converting data into a greater number of services. Yet, since the link between digital footprint and (CX) cannot simply be tied to its availability through data, but rather with organizations’ competency of working with it, this study brings an interpretative framing to the literature on digital footprint and (CX)s by concentrating on analytical capabilities without implicitly demanding such a demarcation of what part of the plethora of digital data can serve value in service.

However, at a higher layer of application, one can derive from the findings of this study that internet services is playing role not just by collecting digital data on customers but also developing itself to have improved capability of making use of this data to tailor service design, offerings and responses to meet customer requirements. Managers of these (internet service provider) companies are basically classified as those who: scrutinize the further development of infrastructure inside the community, assess data and investigate its relationship to utility choices in necessary contexts and build an internal common view that integrates digital identity, privacy safety, and trust.

The importance of this study is that it deals with the internet services sector in Nineveh Governorate, located in a developing environment such as Iraq and the maturity of this environment for simultaneous digital interactions while at the same time facing the challenges of electronic teamwork and localization. As such, the significance of the study is not to define a global phenomenon so much as to show how this museum-developmental phenomenon plays out in a locality that it still building both capacity and analysis. As a result, this study provides a more contextualized insight into the use of digital footprints to optimize (CX) in a service sector that is tied to daily digital engagement.

Fortunately, you have internet services companies using their data analytical powers essentially to create a product out of the digital information and then sell it back to consumers in good measure (the sales part nowadays at least), enabling customers to make personal decisions based on helpful advice. This means connecting digital data to services, personalizing it and responding according to customer requirements, this means building newer practices in the organization itself and innovating with techniques from the data perspective. This study also shows the need to reconcile the forces that animate these corporations.

### 5.2 Limitations and future research

The results of the current study must be interpreted in light of some limitations. Data collected using questionnaires, as in this study, could be affected by self-reporting bias and variations in the digital maturity of respondents. The major limitation, however, is that (CX) was only assessed from the ISP-side perspective because no data were collected from the customer side. The (CX) is too complex to be adequately represented at this granularity. To further elaborate, future work may use dual-perspectival designs that triangulate data from service providers and service customers to form a deeper understanding of how digital footprints relate to (CX). Future research can also widen the regional analysis through comparisons among Iraqi governorates, or between settings with differing digitization maturity. Furthermore, the sophisticated models may be examined through including mediating and/or moderating variables like trust, privacy and analytics capability in an attempt to provide better understanding of mechanisms on how digital footprints might improve customer-experience.

## Ethical approval

At the time we conducted our study, our institution (the University of Mosul, Iraq) did not have an Institutional Review Board (IRB) or an official ethics committee appointed to scrutinize social science research. For this reason, gaining formal ethical approval was not practical. However, we followed commonly accepted ethical standards of studies with human subjects. Participation was partly voluntary, informed consent collected electronically before the first page of the questionnaire, no personal identification data were collected and confidentiality/anonymity fully respected during study.

## Data Availability

-
**Primary Data:** The primary data supporting the findings of this study were collected using a structured survey questionnaire and analyzed using SPSS software. The dataset includes respondents’ demographic information (gender, age, and educational level) as well as responses to the study variables measured on a five-point Likert scale. Due to ethical considerations and data protection requirements, the raw dataset (SPSS file) is not publicly available but is available from the corresponding author upon reasonable request and in accordance with ethical guidelines. -
**Extended Data:** The extended data consist of the survey questionnaire used for data collection. This file has been publicly deposited in the Zenodo repository under a Creative Commons CC0 1.0 Universal dedication and can be accessed at the following DOI: https://doi.org/10.5281/zenodo.17989147 ( Al-Jubory et al., 2025). The corresponding author may provide additional materials upon reasonable request and in compliance with ethical and data protection principles. Corresponding author email: mohamed_almola@uomosul.edu.iq
