# Supplementary material for: Digital Footprint and Its Impact on Improving Customer Experience: An Exploratory Study of a Sample of Internet Companies in Nineveh Governorate
Source: F1000Res. 2026 Apr 30;15:116. Originally published 2026 Jan 24. [Version 2] doi: 10.12688/f1000research.174437.2 (PMC13153770; doi:10.12688/f1000research.174437.2)
Supplement: Peer review history

# Reviewer report: Reviewer response for version 2

**DOI:** 10.5256/f1000research.198227.r480583

**Published:** 2026-05-07

**Authors:** Janet Muthimi

**Affiliations:** 1 Kenyatta University, Nairobi, Kenya

I have reviewed the new version and would like to mention that the revisions are appropriate and sufficient for me.

Is the work clearly and accurately presented and does it cite the current literature?

Partly

If applicable, is the statistical analysis and its interpretation appropriate?

Partly

Are all the source data underlying the results available to ensure full reproducibility?

Partly

Is the study design appropriate and is the work technically sound?

Partly

Are the conclusions drawn adequately supported by the results?

Partly

Are sufficient details of methods and analysis provided to allow replication by others?

Partly

Reviewer Expertise:

Strategic Management, entrepreneurship and leadership

I confirm that I have read this submission and believe that I have an appropriate level of expertise to confirm that it is of an acceptable scientific standard.

# Reviewer report: Reviewer response for version 2

**DOI:** 10.5256/f1000research.198227.r481609

**Published:** 2026-05-07

**Authors:** Rakibul Hasan Chowdhury

**Affiliations:** 1 Trine University, Angola, Indiana, USA

Full Reviewer Report

 �� Summary of the Article

 This manuscript investigates the role of digital footprints in enhancing customer experience (CX) within Internet Service Providers (ISPs) in Nineveh Governorate, Iraq. Using a quantitative approach based on survey data collected from 319 ISP employees and managers, the study examines the relationship between digital footprint utilization and perceived improvements in customer experience.

 The findings reveal a statistically significant positive relationship between digital footprint practices and CX enhancement. However, the study also identifies a critical gap between data collection and the effective analytical use of such data, highlighting the importance of organizational capabilities in transforming digital data into actionable insights.

 The manuscript contributes to the literature by addressing a **contextual gap in developing economies**, particularly in the Iraqi ISP sector, where empirical evidence remains limited.

 �� Detailed Evaluation and Justification

 1. Clarity of Presentation and Use of Literature — **Partly**

 The manuscript is generally well-structured and demonstrates a clear research focus. The introduction effectively establishes the relevance of digital footprints and customer experience in contemporary digital environments. The discussion section is particularly strong in linking findings to prior studies.

 However, several issues remain:

- Some sections contain **redundant or overly descriptive language**, reducing clarity.
- The manuscript primarily **confirms existing literature** rather than extending it theoretically.
- While recent references are included, the study would benefit from incorporating **more foundational theoretical frameworks** (e.g., customer journey models, service-dominant logic).

✅ **Required improvement:**

- Refine language for conciseness and precision
- Strengthen theoretical positioning beyond literature confirmation

2. Study Design and Technical Soundness — **Partly**

 The study design (cross-sectional survey) is appropriate for exploratory research. The sample size (n = 319) is adequate, and ethical considerations are addressed.

 However, technical limitations reduce robustness:

- The study relies on a **single-source dataset (ISP-side only)**, introducing potential bias
- The conceptual model is **overly simplified**, assuming a direct relationship without considering mediating or moderating variables
- Measurement reliability for the digital footprint construct is **borderline acceptable (Cronbach’s alpha = 0.661)**

✅ **Required improvement:**

- Acknowledge and discuss potential **common method bias more rigorously**
- Justify construct reliability or refine measurement items
- Expand conceptual justification of the model

3. Replicability of Methods — **Partly**

 The manuscript provides a reasonable description of:

- Sampling approach
- Questionnaire structure
- Statistical techniques

However, full replication is limited due to:

- Absence of **detailed measurement items within the manuscript**
- Lack of **advanced validation procedures** (e.g., confirmatory factor analysis)
- Limited transparency in **data preprocessing and analytical steps**

✅ **Required improvement:**

- Provide clearer details on measurement scales and item sources
- Include validation procedures or justify their absence
- Clarify analytical workflow for reproducibility

4. Statistical Analysis and Interpretation — **Partly**

 The statistical methods used (correlation and simple linear regression) are appropriate for exploratory analysis and are correctly interpreted.

 However:

- The use of **simple linear regression is insufficient** for capturing the complexity of customer experience
- The model explains only **39% of variance (R² = 0.39)**, indicating missing explanatory variables
- No robustness checks or alternative models are tested

✅ **Required improvement:**

- Acknowledge model limitations more explicitly
- Suggest or incorporate more advanced analytical approaches (e.g., multiple regression, SEM)

5. Data Availability and Reproducibility — **Partly**

 The study provides:

- A clear description of the dataset
- Access to the questionnaire via a public repository

However:

- The **raw dataset is not publicly available**, limiting reproducibility
- Data access is conditional (“upon reasonable request”), which reduces transparency

✅ **Required improvement:**

- Provide stronger justification for restricted data access
- Where possible, consider anonymized dataset sharing

6. Validity of Conclusions — **Yes**

 The conclusions are **generally well-supported by the results**.

- The identification of a **positive relationship between digital footprints and CX** is consistent with the data
- The recognition of a **data-utilization gap** is a valuable and evidence-based insight

Importantly, the authors appropriately avoid overgeneralization and acknowledge limitations, which strengthens the credibility of the conclusions.

 ⚠️ Key Issues That Must Be Addressed (Mandatory Revisions)

 To ensure the manuscript is **scientifically sound**, the following points must be addressed:

 �� Major Revisions Required

1. **Improve measurement validity**
  - Address low Cronbach’s alpha
  - Clarify scale development and validation
2. **Strengthen methodological rigor**
  - Discuss and mitigate common method bias
  - Provide clearer analytical procedures
3. **Enhance conceptual model**
  - Justify the direct relationship assumption
  - Acknowledge missing variables (e.g., trust, privacy, analytics capability)

�� Minor but Important Revisions

1. Improve clarity and reduce redundancy in writing
2. Strengthen theoretical contribution beyond literature confirmation
3. Provide more transparency in data handling and availability

�� Final Recommendation

**✔ Approved with Reservations**

 The manuscript addresses an important and underexplored topic and provides valuable contextual insights into digital transformation and customer experience in a developing economy. With the suggested revisions, it has strong potential to make a meaningful contribution to the literature.

Is the work clearly and accurately presented and does it cite the current literature?

Partly

If applicable, is the statistical analysis and its interpretation appropriate?

Partly

Are all the source data underlying the results available to ensure full reproducibility?

Partly

Is the study design appropriate and is the work technically sound?

Partly

Are the conclusions drawn adequately supported by the results?

Partly

Are sufficient details of methods and analysis provided to allow replication by others?

Partly

Reviewer Expertise:

Digital TransformationCustomer Experience Management (CXM)Data Analytics & Business IntelligenceDigital Marketing & PersonalizationInformation Systems & Enterprise TechnologyBig Data AnalyticsService InnovationTechnology Adoption in Emerging MarketsPlatform Economy & Digital EcosystemsAI-Driven Customer Insights

I confirm that I have read this submission and believe that I have an appropriate level of expertise to confirm that it is of an acceptable scientific standard, however I have significant reservations, as outlined above.

# Reviewer report: Reviewer response for version 1

**DOI:** 10.5256/f1000research.192339.r463404

**Published:** 2026-03-26

**Authors:** Janet Muthimi

**Affiliations:** 1 Kenyatta University, Nairobi, Kenya

**Overall Verdict**

**MAJOR REVISION REQUIRED**

 This paper explores a concept that is of practical significance-the use of customer digital trails to improve CX among ISPs operating in a developing country setting. With respect to context, Nineveh Governorate, Iraq is not an area that is widely studied in terms of digital marketing and CX, so there is the possibility for valuable context contribution here.

 That being said, as it stands now the paper has serious issues with regard to research methodology, organization, and analysis. It is not yet at a point where it could be considered for indexing in a globally peer reviewed journal because it does not meet scientific writing standards. By this I do not mean minor grammatical errors or stylistic critiques. There are issues within the manuscript that put the integrity, dependability, and reproducibility of your results into question. 

 The sections below document specific concerns under each major component of the paper.

**1. Title and Abstract**

**1.1 Strengths**

 • The title effectively conveys the study's main focus, the key variables involved (digital footprint and customer experience), and the specific context (Nineveh Governorate), fulfilling fundamental indexing criteria.

 • The selected keywords, including Digital Footprint, Customer Experience, Personalization, Internet Service Providers, and Nineveh, are largely suitable and relevant to the study's scope.

**1.2 Weaknesses and Required Revisions**

- The Background section of the abstract is wordy and repetitive. The main idea that ISPs underutilize digital footprint data is repeated at least three times, using slightly different wording. The abstract should clearly present the research gap, methodology, and main findings in a concise manner, within 250–300 words.
- The Methodology section of the abstract is too brief. It only mentions the sample size (n = 319) and the type of tool used (structured questionnaire). There is no information about the analytical techniques (such as Pearson correlation, simple linear regression, or the structural equation model shown in Figure 2), the sampling method, or the unit of analysis. A complete methodology statement in an abstract should include all of these details.
- The Results section mixes a managerial suggestion with a scientific result. The statement "This, in turn, suggests a need to strengthen the analysis capacity of service providers" is a recommendation, not a reported finding. Abstracts should only summarize what was discovered, not what should be done next.
- The term "Nineveh" as a single geographic term has limited usefulness in academic searches. Using "Iraq" or "developing countries" instead would greatly improve the chances of the paper being found in different databases.

**2. Introduction**

**2.1 Strengths**

- The introduction appropriately situates the study within the broader CX and digital data literature by referencing established foundational works (e.g., Lemon & Verhoef, 2016; Wedel & Kannan, 2016).
- The three-point research objective list at the close of the introduction provides reasonable directional clarity and the acknowledgment of ethical and strategic tensions in digital data use reflects awareness of the scholarly debate.

**2.2 Weaknesses and Required Revisions**

- The introductory paragraph combines organizational transformation, digital data strategy, and competitive advantage without a clear logical progression. The transition from a general idea of organizational change to the specific concept of digital footprints is abrupt and lacks adequate theoretical support.
- The claim that "individuals' virtual-digital footprints are more valuable than any other commodity" (Hassan, 2024) is an exaggerated and unfalsifiable statement that is not appropriate for inclusion in an academic introduction. This statement should either be removed or replaced with a more balanced, evidence-based assertion.
- The identification of a research gap is presented rather than supported. Statements such as "research remains limited" and "has not been thoroughly investigated" require systematic validation. For example, using a structured database search (Scopus, Web of Science) to demonstrate a lack of studies focusing on digital footprints within the context of Internet Service Providers (ISPs) in Iraq or similar developing countries.
- There is a fundamental mismatch between the stated research objectives and the methodology used. Objective 2 refers to "customer awareness," yet the survey is exclusively directed at ISP employees or their representatives. Therefore, the study cannot legitimately draw conclusions about customer awareness. Research objectives must align with the actual unit of analysis.
- A clearly defined research question is notably absent, which is a standard methodological requirement in exploratory quantitative research. A specific research question should be clearly stated before presenting the hypotheses.
- Section 1.1 - Study Problem: contains a grammatical inconsistency: "it can be a driver of personalized customer experiences on the one hand, and a possible cause on the one hand, and via privacy and trust concerns on the other." The phrase "on the one hand" is used redundantly, and the sentence structure is incomplete. This issue requires correction.
- Section 1.2 - Importance of the Study: outlines three advantages of digital footprint analysis that are broadly applicable across any sector and any geographical location. No specific justification is provided to explain why this study holds particular significance within the Nineveh/Iraqi context. There is also a lack of references to local regulatory frameworks, Iraq's national digital transformation strategy, or regional economic indicators.
- Objective 3 - Provide recommendations to businesses: is a practitioner-oriented outcome rather than a legitimate scientific research objective. It should be rephrased as an investigative goal, for example, "Identify evidence-based strategies for the effective and ethical use of digital footprints within local ISP contexts."

**3. Literature Review and Conceptual Framework**

**3.1 Strengths**

- The review draws on a reasonably broad range of sources, including foundational texts (Lemon & Verhoef, 2016; Zuboff, 2019), domain-specific studies (Koidl et al., 2018; Micheli et al., 2018), and more recent contributions (Neelam et al., 2025; Hardcastle et al., 2025).
- Table 1, which summarizes advantages and disadvantages of digital footprints across four dimensions, provides a structured overview that is useful to the reader.

**3.2 Weaknesses and Required Revisions**

- Section 2.1 begins with an Arabic linguistic analysis of the word "footprint," as defined by Ibn Manzur (1998), a respected classical Arabic dictionary. While this linguistic exploration offers cultural insight, it is not directly relevant to the study's scientific goals and should either be removed or significantly shortened. The claim that digital footprint generation was "expected to increase 44 times by 2020" (Arya et al., 2019) is a prediction that has already passed. The manuscript should either confirm the actual results or replace this claim with a more recent and well-supported data source.
- Section 2.2 contains an important methodological inconsistency in labeling. The section title correctly refers to the two categories as "active and passive" digital footprints; however, the passive category is inconsistently referred to as "negative Digital Footprints" throughout the text. This terminology issue needs to be corrected throughout the manuscript. In Table 1 (Section 2.2), the citation in the Social Interaction row (Sorqvist & Holmgren, 2022) is incorrectly used. That publication addresses methods for estimating environmental impact, particularly the "negative footprint illusion" in sustainability research, and is unrelated to ethical concerns about customer profiling in digital marketing. An appropriate reference should be substituted.
- Section 2.3 - Table 1: outlines four aspects of digital footprint (personalization, user experience, social interaction, location-based services) without specifying the theoretical or empirical basis for choosing these dimensions over other classifications found in existing literature. The selection criteria should be clearly explained. There is a structural difference between the four customer experience (CX) dimensions mentioned and the four pathways shown in Figure 1, which causes a lack of alignment and undermines the conceptual framework. These two sets of dimensions need to be aligned or the model may require reorganization.
- Figure 1 is overly schematic. It displays digital footprint influencing four unlabeled boxes that collectively impact customer experience, but it provides no explanation of causal relationships, mediating factors, or moderating conditions. This representation is not a testable structural model but rather an abstract conceptual diagram. It should be redesigned based on theoretical foundations and with clear directional logic.
- The three hypotheses at the end of Chapter 2 are poorly formulated. Hypothesis 1 states "statistically significant differences in respondents' perceptions," which is a descriptive hypothesis that does not match the regression analysis used later in the manuscript. Hypotheses 2 and 3 are presented in null form, which is not common in quantitative business research, where the alternative hypothesis is usually stated. All three hypotheses should be rewritten in a positive, directional format that aligns with the analytical approach used.

**4. Methodology**

**4.1 Strengths**

- The authors transparently acknowledge the absence of a formal Institutional Review Board at the University of Mosul and clearly describe the compensatory ethical measures applied (electronic informed consent, voluntary participation, data anonymity). This is appropriate for the institutional context.
- The use of a five-point Likert scale and structured questionnaire is a reasonable methodological choice for this type of exploratory quantitative study.

**4.2 Weaknesses and Required Revisions**

- The sampling methodology described is lacking in detail. The manuscript does not clarify whether the 319 participants were chosen through random, purposive, or convenience sampling; the methods used to identify and involve Internet Service Providers (ISPs) in the Nineveh Governorate; the response rate; or if a formal sample size determination (such as power analysis, Krejcie and Morgan table, or G*Power estimation) was performed. These elements are crucial for assessing the representativeness and generalizability of the study's findings.
- The unit of analysis is not clearly defined. Although the study population is described as "Internet service companies operating within Nineveh Governorate," Table 2 indicates that the respondents are adult individuals with varied demographic backgrounds. The study does not specify whether these individuals are managers, employees, or customers of ISPs, which is a significant flaw that undermines the analytical framework's integrity.
- The questionnaire instrument is insufficiently described. The manuscript fails to mention the total number of items, the specific constructs measured in each section, the scale anchors used, whether items were sourced from validated instruments in existing literature, or if the instrument underwent content validity evaluation (such as expert panel review or content validity index). This information is essential for replicating the study.
- No reliability analyses are provided. It is crucial to calculate and report Cronbach's alpha or McDonald's omega for each latent construct, especially given the use of multi-item Likert scales. The absence of any reported reliability coefficient is a major shortcoming in validating the measurement instruments.
- No validity assessments are mentioned. It is necessary to conduct and report confirmatory or exploratory factor analysis, or at least evaluations of convergent and discriminant validity, particularly since Figure 2 appears to depict a structural equation model.
- The choice of Pearson's correlation coefficient and ordinary least squares regression is not theoretically justified given the ordinal nature of Likert-scale data. The authors should either use Spearman's rho for correlation analysis and explain their regression methodology or explicitly justify treating aggregated Likert scores as interval data in this context.
- Common Method Bias (CMB) poses a significant and well-documented risk, especially when both the independent variable (digital footprint perceptions) and the dependent variable (customer experience perceptions) are collected through self-report from the same respondents in a single survey. The manuscript does not address this potential risk, nor does it implement any procedural or statistical remedies (such as Harman's single-factor test, marker variable technique, or temporal separation of measures).

**5. Results**

**5.1 Strengths**

- The descriptive statistics in Tables 3 and 4 provide a reasonably clear summary of central tendency and dispersion for each item.
- The correlation coefficient (r = 0.60, p < 0.001) and regression coefficient (β = 0.826, R² = 39%) are clearly reported alongside their significance levels, which supports transparency.

**5.2 Weaknesses and Required Revisions**

- Section 4.1 describes the sample as "mature" solely because most respondents are males aged 31–50. This description is an unsupported inferential assumption. In research methodology, maturity is typically linked to professional experience or expertise in a field, rather than just age. Therefore, the description should be revised to provide an objective demographic summary.
- The questionnaire items listed in Tables 3 and 4 are labeled only as x1–x15 and y1–y20, without indicating the specific constructs they measure. Without the content of these items, readers cannot determine if the reported means and standard deviations accurately reflect the intended constructs, nor can they assess face validity or replicate the measurement tool.
- Figure 2 shows a structural equation model with latent variables (XX, YY, AA, BB, CC, EE) and standardized path coefficients. However, the methodology section does not mention the use of Structural Equation Modeling (SEM). If SEM was used, it is essential to report standard model fit indices (CFI, TLI, RMSEA, SRMR, chi-square/df ratio). If Figure 2 is merely a conceptual diagram not related to statistical analysis, it should be removed or clearly labeled as such to prevent misunderstanding.
- Hypothesis 1, which claims statistically significant differences in respondents' perceptions across various variables, is evaluated only through descriptive statistics (means, standard deviations, agreement percentages). No inferential statistical tests (such as t-tests, one-sample tests against the midpoint of 3, or Mann-Whitney U tests) are mentioned. As a result, the criteria for accepting or rejecting this hypothesis are not specified.
- The coefficient of determination (R² = 39%) indicates that the digital footprint variable explains about 39% of the variance in customer experience. The remaining 61% is vaguely attributed to "random variables that could not be controlled." This explanation is methodologically insufficient. The discussion should delve into the theoretical implications of the unexplained variance, potential mediating or moderating variables, and the limitations of using a single-predictor model.

**6. Discussion, Conclusion, and Limitations**

**6.1 Strengths**

- The authors appropriately acknowledge the analytical capability gap among ISPs as a barrier to translating data collection into actionable customer experience improvements, which aligns with the implementation-gap literature (cf. Koidl et al., 2018).
- The recommendation to adopt balanced, evidence-based, and privacy-respecting data strategies is practically relevant to ISPs operating in nascent digital regulatory environments.

**6.2 Weaknesses and Required Revisions**

- The discussion section is conspicuously concise (approximately 350 words) in relation to the intricate nature of the statistical results. This discussion fails to sufficiently engage with the theoretical ramifications of the results. This section must be augmented to a minimum of 800–1,000 words. The primary finding that Internet Service Providers (ISPs) accumulate digital data yet exhibit a lack of analytical sophistication to leverage it is posited as a novel contribution. Nevertheless, this phenomenon has been extensively documented in the global literature (Koidl et al., 2021; Koidl et al., 2018). The authors are required to delineate what is uniquely significant about this finding within the Nineveh/Iraqi ISP context to substantiate its academic significance.
- Section 5.1 – Conclusion: reiterates the results rather than synthesizing their broader theoretical and practical implications. There is a lack of discourse on how the findings extend, challenge, or qualify existing theoretical frameworks (e.g., the experience economy model, privacy calculus theory, or data-driven customer experience management theory). Theoretical contributions require explicit articulation. Despite Objective 3 distinctly committing to the provision of recommendations for businesses, this study concludes without any organized managerial recommendations. This represents an unfulfilled commitment that should be rectified in a dedicated section.
- The limitations section acknowledges the utilization of questionnaire-based data collection and the variability in respondents' digital maturity; however, it neglects the most critical limitation of the study: the total exclusion of the customer perspective. A study purporting to examine "customer experience" should ideally incorporate data from customers or, at the very least, clearly acknowledge and theoretically justify exclusive reliance on the supply-side (ISP) perspective. This omission should be explicitly stated and addressed. This study does not delineate specific avenues for future research that logically stem from its findings.
- Future research directions should be rooted in the identified gaps, for instance, multi-level studies integrating ISP and customer perspectives, comparative studies across regions with varying levels of digital maturity, or longitudinal studies monitoring the influence of data governance enhancements on customer experience outcomes.

**7. Summary Assessment**

**Criterion**

**Rating**

 Clarity and accuracy of presentation

 Partly

 Appropriateness of study design

 Partly

 Sufficiency of methodological detail for replication

 No

 Appropriateness of statistical analysis and interpretation

 Partly

 Availability of source data for reproducibility

 Partly

 Adequacy of support for conclusions drawn

 Partly

 Citation of current and relevant literature

 Partly

 Overall Recommendation

 MAJOR REVISION REQUIRED

 The paper reflects a legitimate attempt to investigate a contextually under-explored question. Nevertheless, the extent and gravity of the identified deficiencies, particularly regarding instrument transparency, reliability and validity reporting, clarity of the unit of analysis, hypothesis construction, and the absence of customer-side data, necessitate substantial revision before the manuscript can meet the standards required for international scholarly indexing. A comprehensive resubmission that addresses all issues raised in this report would be welcomed.

Is the work clearly and accurately presented and does it cite the current literature?

Partly

If applicable, is the statistical analysis and its interpretation appropriate?

Partly

Are all the source data underlying the results available to ensure full reproducibility?

Partly

Is the study design appropriate and is the work technically sound?

Partly

Are the conclusions drawn adequately supported by the results?

Partly

Are sufficient details of methods and analysis provided to allow replication by others?

Partly

Reviewer Expertise:

Strategic Management, entrepreneurship and leadership

I confirm that I have read this submission and believe that I have an appropriate level of expertise to confirm that it is of an acceptable scientific standard, however I have significant reservations, as outlined above.

# Response

**Published:** 2026-03-30

**Authors:** saif zakaria

**Affiliations:** Management Information Systems, University of Mosul, Mosul, Nineveh Governorate, Iraq

**Response to Reviewer 2**

 We would like to express our sincere appreciation to Reviewer 2 for the careful reading of our manuscript and for the constructive and insightful comments provided. We highly value the reviewer’s time, effort, and scholarly observations, which helped us improve the clarity, coherence, methodological transparency, and overall academic quality of the paper. In revising the manuscript, we made every effort to address the comments as fully as possible and to align the revised version with the reviewer’s recommendations. Our point-by-point responses are presented below.

**1. Title and Abstract**

**Comment 1.1**

**Response: **Thank you for this valuable comment. The abstract background was revised to make it more concise and focused on the research gap. In the revised version, the background now states more directly that although ISPs collect and build customers’ digital traces, evidence remains limited regarding how such data are utilized for customer-experience enhancement in Iraqi ISP contexts. This revision was intended to reduce repetition and improve clarity.

**Comment 1.2**

**Response: **We appreciate this important observation. In the revised abstract, we expanded the methodology to specify the respondents, the unit of analysis, the measurement scale, and the analytical techniques used. The abstract now states that the data were collected through an online structured questionnaire from 319 employees and managers in ISPs in Nineveh Governorate/Iraq, that the individual respondent was used as the unit of analysis, and that descriptive statistics, reliability analysis, correlation tests, and simple linear regression were used.

**Comment 1.3**

**Response: **Thank you for this comment. The results statement in the abstract was revised to focus more clearly on the empirical findings. The revised version now reports that digital footprints significantly support service personalization and operational efficiency and that there is an implementation gap between data collection and the effective use of data for creating value through customer interactions. While the abstract conclusion still retains a practical orientation, the results portion itself was made more clearly evidence-based.

**Comment 1.4**

**Response: **We appreciate this suggestion. In the revised manuscript, the study is framed more explicitly within the Iraqi and developing-context setting through the abstract, introduction, and study-importance section, which strengthens contextual visibility and retrieval beyond the local label alone.

**2. Introduction**

**Comment 2.1**

**Response: **Thank you for this observation. The introduction was reorganized to improve logical progression from technological change and customer centricity to customer experience, internet-based services, and digital footprints. In the revised version, the opening paragraph now establishes a clearer conceptual link between these elements.

**Comment 2.2**

**Response: **We appreciate this important point. The revised introduction avoids the earlier exaggerated formulation and instead states that digital footprints provide firms with valuable behavioral insights and have growing strategic importance because they support personalization, service improvement, and customer-experience outcomes. This change was made to ensure a more balanced academic tone.

**Comment 2.3**

**Response: **Thank you for this comment. In response, we added a paragraph explaining that a preliminary structured search was conducted in major academic databases, including Scopus and Web of Science, using keywords related to digital footprints, customer experience, and ISPs. The revised manuscript now states that the existing literature is concentrated in developed economies and that limited attention has been given to ISP-related digital-footprint studies in developing contexts, particularly Iraq.

**Comment 2.4**

**Response: **We appreciate this valuable observation. The study aims were revised in order to better reflect the analytical logic of the paper and the ISP-side perspective of the study. In the revised manuscript, the aims focus on the use of digital-footprint data in ISP practices, the deployment of such data in enhancing customer experience, and the relationship and impact between digital footprints and customer experience. This was intended to strengthen alignment between the study objectives, hypotheses, and methodology.

**Comment 2.5**

**Response: **We fully agree with the reviewer on this point. In the revised manuscript, the wording was adjusted to make clear that the study evaluates customer-experience enhancement from the perspective of ISP personnel rather than from customers directly. This clarification now appears explicitly in the abstract and is supported in the methods section through the definition of the respondents and unit of analysis.

**Comment 2.6**

**Response: ** Thank you for highlighting this issue. The study-problem section was rewritten in the revised manuscript. The revised text now explains the dual nature of digital footprints more clearly, namely that they may support personalization while also raising privacy- and trust-related concerns, without the previous grammatical inconsistency.

**Comment 2.7**

**Response: **We appreciate this important observation. The study-importance section was revised to incorporate context-specific justification. In the revised version, we added references to the expansion of the digital environment in Iraq, the institutional assessment of digital maturity in e-governance, and the particular relevance of the ISP sector as one of the sectors most directly connected to users’ digital interactions in Nineveh and Iraq.

**Comment 2.8**

**Response: **Thank you for this comment. Objective 3 was revised in a more research-oriented direction. In the revised manuscript, it now refers to identifying evidence-based strategies for the effective and ethical use of digital footprints within local ISP contexts, rather than simply “providing recommendations.”

**Comment 2.9**

**Response: **We appreciate this suggestion. In the revised manuscript, the research problem was reformulated in question form under Section 1.1 to function more explicitly as the guiding research question of the study.

**3. Literature Review**

**Comment 3.1**

**Response: **Thank you for this suggestion. The revised manuscript no longer opens the conceptual discussion with the earlier Arabic etymology. Instead, the section begins with a more direct academic treatment of the concept of digital footprint and traces its development in the literature.

**Comment 3.2**

**Response: **We appreciate this point. In the revised version, this projection is no longer emphasized. Instead, the literature review discusses the growing importance of digital footprints through references to increased internet use, social-media adoption, mobile technologies, and smart-device interaction.

**Comment 3.3**

**Response: **Thank you for this important observation. The terminology was standardized in the revised manuscript. The two categories are now presented consistently as active and passive digital footprints.

**Comment 3.4**

**Response: **We thank the reviewer for identifying this issue. In the revised manuscript, the problematic citation was removed from the discussion of passive digital footprints and replaced with more appropriate sources, including Estrada-Jiménez et al. (2017), in relation to passive data, profiling, and digital-marketing uses.

**Comment 3.5**

**Response: **In the revised manuscript, Table 1 is retained as a structured overview of selected implications of digital footprints rather than as a formal multidimensional causal model. The broader tested model was simplified so that these aspects are no longer presented as separate causal pathways.

**Comment 3.6**

**Response: **Thank you for this important point. Figure 1 was revised and simplified in the new version of the manuscript so that it now presents a direct relationship between digital footprints and customer experience, rather than four separate pathways. This revision was made to improve coherence between the literature review, the conceptual model, and the analytical design used in the study.

**Comment 3.7**

**Response: **The conceptual model was revised to present a clearer and more testable form. The revised version now assumes a direct positive relationship between digital-footprint use and perceived customer-experience improvement, supported by a short theoretical rationale before the hypotheses.

**Comment 3.8**

**Response: **The hypotheses were reformulated in a positive and directional manner. In the revised manuscript, the model now includes two hypotheses: one concerning the positive association between digital-footprint use and perceived customer-experience improvement, and another concerning the positive and statistically significant effect of digital-footprint use on perceived customer-experience improvement. This revision was made to align the hypotheses with the correlation and regression analyses actually used in the study.

**4. Methods**

**Comment 4.1**

**Response: **The revised manuscript retained and clarified the informed-consent procedure, voluntary participation, confidentiality assurances, and the explanation regarding the institutional context in which the study was conducted.

**Comment 4.2**

**Response: **In the revised methodology, we added a clearer explanation of how the study population and sample were identified. Specifically, the revised manuscript states that Nour Al-Badia was identified as the principal ISP in Nineveh Governorate, with 58 subsidiary service providers under its network, and that a questionnaire link was sent to a sample of 390 individuals, from whom 319 responses were collected. While the revised manuscript improves transparency substantially, we acknowledge that the sampling technique is not yet expressed in formal probabilistic terminology.

**Comment 4.3**

**Response: **The revised manuscript now reports that the questionnaire contained 35 items distributed across two variables: customer digital footprint (15 items) and customer experience (20 items), measured through a five-point Likert scale. The revised version also explains that some items were adapted from previous tools while others were developed to fit the Mosul context, and that content validity was reviewed by digital-marketing experts.

**Comment 4.4**

**Response: **The revised abstract explicitly states that the individual respondent was used as the unit of analysis, and the methods section now clarifies that the respondents were employees and managers working in the ISP sector. This was intended to resolve the earlier ambiguity about whether the study concerned organizations, customers, or individual respondents.

**Comment 4.5**

**Response:** In the revised manuscript, Cronbach’s alpha was added and reported for the main constructs. The revised text now includes reliability information for the digital-footprint items and for the customer-experience variable. This addition was made directly in response to the reviewer’s concern about internal consistency.

**Comment 4.6**

**Response: **In the revised manuscript, content validity was addressed through expert review of the questionnaire items. At the same time, the revised version explicitly states that no convergent or discriminant validity assessments and no confirmatory factor analysis were conducted. Rather than leaving this implicit, we presented it transparently as a limitation and as a direction for future instrument development.

**Comment 4.7**

**Response: **In the revised version, Pearson’s correlation and simple linear regression are now stated explicitly in the statistical-methods section. While we acknowledge that the methodological justification could be expanded further, the revised manuscript makes the analytical choice transparent and consistent with the treatment of the variables as composite scores based on Likert-scale responses.

**Comment 4.8**

**Response: **In response, the revised manuscript now reports Harman’s one-factor test. The revised text states that the first factor accounted for 28.3% of the total variance, which is below the commonly used 50% threshold, suggesting that common-method bias is less likely to dominate the data.

**5. Results**

**Comment 5.1**

**Response: **In the revised manuscript, the description of the respondents was made more objective by focusing on age, gender, and educational attainment instead of using interpretive labels such as “mature.”

**Comment 5.2**

**Response: **In the revised version, the results tables no longer depend only on x/y coding; instead, the actual questionnaire items are shown in Tables 3 and 4. This was done to make the operational measures more transparent and interpretable.

**Comment 5.3**

**Response: **In the revised manuscript, greater emphasis is placed on the correlation and regression analyses as the core empirical tests of the study. In addition, the conceptual model in Figure 1 was simplified to match the actual analytical design more closely.

**Comment 5.4**

**Response: **We appreciate this methodological concern. In the revised version, the earlier descriptive hypothesis was removed and replaced by two hypotheses that correspond directly to the correlation and regression analyses used in the study. This revision was intended to improve consistency between the stated hypotheses and the actual statistical procedures.

**6. Discussion, Conclusion, and Limitations**

**Comment 6.1**

**Response: **Thank you for this important comment. The discussion was substantially expanded in the revised manuscript. The revised text now addresses the interpretation of the findings, their consistency and divergence with prior literature, the implementation gap between data collection and analytical use, the contextual relevance of the findings in Nineveh/Iraq, and their theoretical and practical implications. This was done to move the discussion beyond a brief summary of results.

**Comment 6.2**

**Response: **In the revised manuscript, greater emphasis was placed on the contextual significance of the study. The revised text highlights that the Iraqi/Nineveh context is characterized by expanding digital interactions alongside uneven digital maturity and institutional readiness, which helps explain why the gap between data collection and effective analytical use has contextual significance in this setting.

**Comment 6.3**

**Response: **Thank you for this valuable comment. The conclusion was rewritten in a more synthetic and structured manner. In the revised version, it now includes a theoretical conclusion concerning the strategic and informational significance of digital footprints, a practical conclusion related to analytical capability and service design in ISPs, and a contextual conclusion regarding the value of the study in Nineveh Governorate and Iraq.

**Comment 6.4**

**Response: **We appreciate this important point. In response, the revised manuscript now includes practical recommendations directed at ISP managers, including strengthening internal analytical capabilities, improving the integration of digital data into service-related decisions, and maintaining a balanced approach that takes privacy and trust into account.

**Comment 6.5**

**Response: **Thank you for this important observation. The revised limitations section now explicitly states that customer experience was assessed only from the ISP-side perspective and that no customer-side data were collected. The revised manuscript also adds future research directions, including dual-perspective ISP/customer studies, cross-governorate comparisons, and models incorporating trust, privacy, and analytics capability as mediating or moderating variables.

# Reviewer report: Reviewer response for version 1

**DOI:** 10.5256/f1000research.192339.r457991

**Published:** 2026-03-05

**Authors:** Margaretha Pink Berlianto

**Affiliations:** 1 Pelita Harapan University, Tangerang, Tangerang, Indonesia

**Title:** Digital Footprint and Its Impact on Improving Customer Experience: An Exploratory Study of a Sample of Internet Companies in Nineveh Governorate

 1. Title and Abstract.

- The background is verbose and repetitive, restating the same point about the underutilization of digital data multiple times in slightly different phrasing.
- The methodology section is critically thin; it states only that 319 respondents completed a structured questionnaire, with no mention of the analytical approach, sampling technique, statistical tools used, or any other aspects of the research method.
- The results conflate a managerial recommendation with a scientific finding ("This, in turn, suggests a need to strengthen the analysis capacity of service providers"). Conclusions in abstracts should summarize what *was found*, not what ISPs *must* do.
- Keywords are acceptable but generic. "Nineveh" as a standalone keyword adds little indexing value; "Iraq" or "developing countries" would be more useful for retrieval.

2. Introduction

- The introduction appropriately situates digital footprints within the broader context of customer experience (CX) literature and acknowledges the competitive pressure facing ISPs. The three-point research objective list at the end provides reasonable clarity of intent.
- The opening paragraph is conceptually sound but poorly structured. It conflates organizational transformation, digital data strategy, and competitive advantage without a clear logical progression.
- The statement that "people's virtual-digital footsteps are more precious than anything" (Hassan, 2024) is an unscientific, hyperbolic assertion that is unsuitable for an academic introduction.
- The research gap is identified but not quantified or substantiated. There is no systematic mapping of the existing literature to precisely identify the gap. Phrases like "still scarce" and "not sufficiently studied" require supporting evidence — for example, a structured review of databases showing a dearth of ISP-focused digital footprint studies in Iraq.
- The three stated research objectives at the end of the introduction (examining data usage, organizational awareness, perceived outcomes) do not fully align with the hypotheses presented later in Chapter 2.
- The aim of the study, point no 2 mentions "organizational awareness and customer awareness," yet the methodology surveys only ISP employees, not customers. This is a fundamental contradiction. Align objectives with hypotheses and methodology.
- Section 1.1 (Study Problem) contains a grammatical error mid-paragraph: "it can be a driver of personalized customer experiences on the one hand, and a possible cause on the other hand". This sentence is incoherent and appears twice with the exact phrase ("on the one hand").
- The importance section (1.2) lists three generic benefits of digital footprint analysis that could apply to virtually any sector in any country. There is no specific justification for why this study is *critical* in Nineveh Governorate or the Iraqi ISP context — no local economic data, no regional policy context, no reference to Iraq's digital transformation agenda.
- Objective 3 ("provide recommendations") is a practitioner output, not a scientific research objective. In an academic paper, objectives should describe what the study investigates, not what deliverables it will produce. Revise Objective 3 to be research-oriented rather than prescriptive.
- There is no explicit research question formally stated, which is a standard requirement in exploratory research design. Rewrite the research problem as an officially stated research question.

3. Literature Review

- Section 2.1 opens with an Arabic linguistic etymology of the word "footprint" (referencing Ibn Manzur, 1998, a classical Arabic dictionary). While culturally interesting, this is academically irrelevant to the study's objectives and consumes space without contributing to the conceptual framework.
- The claim that the scope of digital footprint generation was "expected to increase 44-fold by 2020" (Arya et al., 2019) is a future projection that has already passed. The paper should present what *actually* happened or use more current data.
- Section 2.2 contains a labeling inconsistency. The second type is introduced as "negative Digital Footprints," but the section header says the two categories are "active and passive." The correct term should be "passive," not "negative."
- Section 2.2, the "negative footprint illusion" reference (Sörqvist & Holmgren, 2022) in the social interaction row appears misappropriated — that paper addresses environmental impact estimation, not customer profiling ethics.
- Section 2.3 (Table 1) lists four aspects (personalization, user experience, social interaction, location-based services) but does not justify why these four were selected over others.
- Section 2.5 presents four CX dimensions. However, these dimensions (relational, physical environment, social, and organizational responsiveness) do not clearly correspond to the four pathways shown in Figure 1 (personalization, user experience, social interaction, and location-based services). This creates a structural disconnect between the literature review and the conceptual model.
- Figure 1 is overly simplistic. It shows a digital footprint leading to four boxes that collectively feed into customer experience, but there are no directional indicators for mediation, moderation, or mechanisms. This is not a testable structural model; it is a diagram.
- The three hypotheses presented at the end of Chapter 2 are poorly constructed. Hypothesis 1 states there are "statistically significant differences in respondents' perceptions" — this is a descriptive hypothesis, not a relational or causal one, and does not align with the regression analysis used later. Hypotheses 2 and 3 are stated in the *null* form, which is methodologically unusual in quantitative business research, where the alternative hypothesis is typically stated. Reconstruct hypotheses in positive/directional form consistent with the analytical methods used. Justify the conceptual model with theoretical grounding.

4. Methods

- The ethical considerations regarding informed consent and data confidentiality are adequately addressed, and the acknowledgment of the absence of an IRB is transparent and appropriate for the Iraqi institutional context.
- There is no explanation of the sampling strategy: Was the sample of 319 drawn randomly, purposively, or through convenience sampling? How were ISPs identified and contacted? What was the response rate? Was a sample size justification (e.g., power analysis or Krejcie & Morgan formula) conducted?
- There is no description of the questionnaire instrument: How many items? What constructs were measured? What scale anchors were used? Were scales adapted from validated instruments or developed de novo? If developed by the authors, was content validity (CVI) or expert panel validation conducted?
- The study population is described as "Internet service companies working in Nineveh Governorate," but the unit of analysis is never clarified. Are the 319 respondents company managers, employees, or customers? Table 2 suggests they are adults with varying demographics, but their relationship to the ISPs is never specified. This is a fundamental omission.
- Reliability testing (e.g., Cronbach's alpha) is not reported anywhere in the paper, raising serious questions about the internal consistency of the multi-item scales.
- Validity testing (convergent, discriminant, or construct validity) is absent.
- The choice of Pearson's correlation and simple linear regression is not justified. Given the Likert-scale ordinal data, Spearman's rho might be more appropriate, or the authors should justify why treating the data as interval-level is defensible.
- Common Method Bias — since both independent and dependent variables were collected from the same respondents in a single survey, this is a significant threat that is never acknowledged or addressed.

5. Results

- Section 4.1 characterizes the sample as "mature" solely because most respondents are male and aged 31–50. This is an interpretive leap with no supporting logic. Maturity in research contexts typically refers to experience or knowledge level, not age bracket.
- The item-level variables in Tables 3 and 4 are labeled x1–x15 and y1–y20 with no description of what each item represents. Readers cannot evaluate whether the items adequately operationalize the constructs without knowing the constructs' content.
- The SEM diagram in Figure 2 shows path coefficients and latent variables (XX, YY, AA, BB, CC, EE), suggesting a structural equation model was run — but this is never mentioned in the methodology section. If SEM was used, the fit indices (CFI, RMSEA, SRMR, χ²/df) must be reported. If Figure 2 is simply a visualization tool, its inclusion without explanation is misleading.
- Hypothesis 1, which asks about "statistically significant differences in respondents' perceptions," is tested through descriptive statistics — not an inferential test. No t-test, ANOVA, or chi-square is presented, so it is unclear how this hypothesis is formally "accepted." Provide complete hypothesis testing procedures, including the test statistic, degrees of freedom, and decision rule for Hypothesis 1.

6. Discussion, Conclusion, and Limitations

- The discussion section is notably brief (approximately 350 words) relative to the complexity of the findings. It does not engage deeply with the theoretical implications of the results, nor does it explain *why* there is an implementation gap between data collection and customer experience improvement. Expand the discussion to at least 800–1,000 words.
- The finding that ISPs collect digital data but lack analytical sophistication to leverage it is presented as a novel contribution, yet this is a well-documented phenomenon globally. The authors do not sufficiently articulate what is *unique* about the Nineveh/Iraqi context that makes this finding distinctive.
- The conclusion (Section 5.1) essentially restates the results rather than synthesizing their broader theoretical and practical significance. There is no discussion of the study's theoretical contribution to the digital footprint or CX literature. Please kindly include theoretical implications and practical recommendations for ISP managers.
- The paper ends without managerial recommendations, despite Objective 3 explicitly stating this as a goal. This is an unmet commitment to the reader.
- The limitation regarding questionnaire-based data and "different levels of respondents' digital maturity" is mentioned, but is underdeveloped. The more critical limitation — the absence of customer perspectives despite the study claiming to examine "customer experience" — is never acknowledged.

Is the work clearly and accurately presented and does it cite the current literature?

Partly

If applicable, is the statistical analysis and its interpretation appropriate?

Partly

Are all the source data underlying the results available to ensure full reproducibility?

Partly

Is the study design appropriate and is the work technically sound?

Partly

Are the conclusions drawn adequately supported by the results?

Partly

Are sufficient details of methods and analysis provided to allow replication by others?

Partly

Reviewer Expertise:

marketing, consumer behavior, branding, retailing

I confirm that I have read this submission and believe that I have an appropriate level of expertise to confirm that it is of an acceptable scientific standard, however I have significant reservations, as outlined above.

# Response

**Published:** 2026-03-30

**Authors:** saif zakaria

**Affiliations:** Management Information Systems, University of Mosul, Mosul, Nineveh Governorate, Iraq

We would like to express our sincere appreciation to Reviewer 1 for the careful reading of our manuscript and for the detailed and constructive comments provided. We highly value the reviewer’s effort and thoughtful observations, which helped us substantially improve the clarity, coherence, and overall academic quality of the paper. In revising the manuscript, we made every effort to address the comments as fully as possible and to strengthen the manuscript in line with the reviewer’s recommendations. Our point-by-point responses are provided below.

**1. Title and Abstract**

**Comment 1.1**

**Response: **The abstract background was revised to make it more concise and to focus more clearly on the research gap. In the revised version, the opening of the abstract now states that although ISPs collect customers’ digital traces, limited evidence exists regarding how such data are used for customer-experience enhancement in Iraqi ISP contexts. This revision was intended to reduce repetition and improve clarity.

**Comment 1.2**

**Response: **In the revised abstract, we expanded the methodology by specifying the respondents, the unit of analysis, the measurement scale, and the analytical methods used. The revised abstract now states that data were collected through an online structured questionnaire from 319 employees and managers in ISPs in Nineveh Governorate, that the individual respondent was used as the unit of analysis, and that descriptive statistics, reliability analysis, correlation tests, and simple linear regression were used.

**Comment 1.3**

**Response: **The results portion of the abstract was revised to focus on the empirical finding that digital footprints significantly support personalization and operational efficiency, while also indicating an implementation gap between data collection and effective value-creating use. We acknowledge that the conclusion of the abstract still retains a practical orientation, but the results statement itself has been made more clearly empirical in the revised version.

**Comment 1.4**

**Response:** The revised version repositions the study more explicitly within the Iraqi and developing-context setting throughout the abstract, introduction, and study-importance section. This was intended to strengthen contextual indexing and retrieval beyond the local reference alone.

**2. Introduction**

**Comment 2.1**

**Response: **The introduction was reorganized to improve logical progression from technological change and customer centricity to customer experience, internet-based services, and digital footprints. The revised opening now establishes a clearer link between technological change, CX, and the strategic value of digital footprints in internet-based services.

**Comment 2.2**

**Response:** In the revised introduction, the idea was reformulated in a more academic manner by emphasizing that digital footprints provide firms with valuable behavioral insights and have strategic importance because they support personalization, service delivery improvement, and customer-experience outcomes. This replaced the earlier exaggerated tone.

**Comment 2.3**

**Response:** we added a paragraph stating that a preliminary structured search was conducted in major academic databases, including Scopus and Web of Science, using relevant keywords. The revised manuscript now explains that the existing studies are concentrated in developed economies and that limited attention has been given to digital footprints and customer experience in ISP contexts in developing settings, particularly Iraq.

**Comment 2.4**

**Response: **The objectives were revised to improve alignment with the study design and the ISP-side perspective. In the revised version, the objectives focus on the extent to which ISPs utilize digital-footprint data, the level of deployment of such data in enhancing customer experience, and the relationship and impact between digital footprints and customer experience. This was done to create stronger coherence between the objectives, the hypotheses, and the methodology.

**Comment 2.5**

**Response: **In the revised manuscript, the framing was shifted to the ISP-side perspective. The abstract now explicitly states that customer-experience enhancement was evaluated from the viewpoint of ISP personnel, and the methodology clarifies that the respondents were employees and managers. This revision was made to avoid implying direct customer-side measurement.

**Comment 2.6**

**Response: **The study-problem section was rewritten. The revised version now explains the dual nature of digital footprints more clearly, noting that they may support personalization while also raising privacy and trust concerns, without the earlier grammatical inconsistency.

**Comment 2.7**

**Response: **The study-importance section was revised to include explicit contextual justification. In the revised manuscript, we added three context-specific arguments: the expansion of the digital environment in Iraq, the basic level of digital maturity across e-governance areas, and the particular relevance of the ISP sector as one of the sectors most directly connected to users’ digital interactions in Nineveh and Iraq.

**Comment 2.8**

**Response: **Objective 3 was revised in a more research-oriented form. In the revised manuscript, it now refers to identifying evidence-based strategies for the effective and ethical use of digital footprints within local ISP contexts, rather than simply “providing recommendations.”

**Comment 2.9**

**There is no explicit research question.**

**Response:** In the revised manuscript, the study problem was reformulated more explicitly and presented in question form in Section 1.1. This was intended to function as the formal research question guiding the study.

**3. Literature Review and Conceptual Framework**

**Comment 3.1**

**Response: **The revised manuscript no longer opens this section with the earlier Arabic linguistic etymology. Instead, the section now begins with a more direct academic treatment of the concept and traces the evolution of the term through the relevant literature.

**Comment 3.2**

**Response: **The revised literature review avoids reliance on the outdated projection and instead discusses the growth of digital footprints through more general and current references to digital-platform expansion, mobile industries, social-media adoption, and smart-device use.

**Comment 3.3**

**Response: **The terminology was revised, and the manuscript now consistently refers to the two categories as active and passive digital footprints.

**Comment 3.4**

**Response:** The problematic citation was removed from the revised discussion of passive digital footprints and replaced by more appropriate references, including Estrada-Jiménez et al. (2017), in relation to passive data, profiling, and digital-marketing use.

**Comment 3.5**

**Response:** In the revised manuscript, Table 1 is retained as a structured overview of selected implications of digital footprints rather than as a separate multidimensional causal model. The broader conceptual model was simplified so that these elements are no longer treated as distinct pathways in the tested framework.

**Comment 3.6**

**Response: **In response, Figure 1 was revised and simplified to present a direct relationship between digital footprints and customer experience, rather than four separate pathways. This was done to improve consistency between the conceptual framework and the actual analytical approach adopted in the study.

**Comment 3.7**

**Response:** The conceptual model was redesigned in a more testable form. In the revised version, the model is theoretically grounded as a direct positive relationship between digital-footprint use and perceived customer-experience improvement. This revision was meant to ensure consistency with the hypotheses and the statistical analyses used in the study.

**Comment 3.8**

**Response: **The hypotheses were reformulated in a positive and directional manner. In the revised version, the manuscript now states two hypotheses: one concerning the positive association between digital-footprint use and perceived customer-experience improvement, and another concerning the positive and statistically significant effect of digital-footprint use on perceived customer-experience improvement. A short theoretical rationale was also added immediately before the hypotheses.

**4. Methods**

**Comment 4.1**

**Response: **The revised version retained and clarified the informed-consent procedure, voluntary participation, confidentiality, and the explanation regarding the absence of an institutional IRB at the time of the study.

**Comment 4.2**

**Response: **In the revised methodology, we added a clearer explanation of how the study population and sample were identified. Specifically, the manuscript now states that Nour Al-Badia was identified as the principal internet service provider in Nineveh Governorate, with 58 subsidiary service providers under its network, and that a questionnaire link was sent to a sample of 390 individuals, from whom 319 responses were collected. We acknowledge that the sampling technique is now more explicit than before, although the revised manuscript does not yet frame it in formal probabilistic terminology.

**Comment 4.3**

**Response: **The revised manuscript now states that the questionnaire contained 35 items distributed across two variables, namely customer digital footprint (15 items) and customer experience (20 items), and that a five-point Likert scale was used. The revised version also clarifies that some items were adapted from previous studies, whereas others were developed to fit the local context, and that content validity was reviewed by digital-marketing experts.

**Comment 4.4**

**Response: **We appreciate this important methodological comment. The revised abstract now explicitly states that the individual respondent was used as the unit of analysis, and the methods section clarifies that the respondents were employees and managers working in the ISP sector. This was intended to resolve the earlier ambiguity regarding whether the study concerned organizations, customers, or individual respondents.

**Comment 4.5**

**Reliability testing is absent.**

**Response:** In the revised methodology, Cronbach’s alpha was added and reported for the main constructs. The revised version presents reliability information for the digital-footprint items and the customer-experience variable. This was added in direct response to the reviewer’s concern regarding internal consistency.

**Comment 4.6**

**Response: **In the revised manuscript, content validity was addressed through expert review of the questionnaire items. The revised text also explicitly acknowledges that no convergent or discriminant validity assessment and no confirmatory factor analysis were conducted. Rather than leaving this issue implicit, we stated it clearly and presented it as an important limitation and an area for future measurement development.

**Comment 4.7**

**Response: **In the revised manuscript, Pearson’s correlation and simple linear regression are now stated explicitly in the statistical-methods section. While we acknowledge that a fuller methodological justification could be developed further, the revised version makes the analytical choice transparent and aligns it with the composite treatment of the study variables based on Likert-scale responses.

**Comment 4.8**

**Response:** In response, the revised manuscript now reports Harman’s one-factor test. The revised text states that the first factor accounted for 28.3% of the total variance, which is below the commonly used 50% threshold, suggesting that common-method bias is less likely to dominate the data.

**5. Results**

**Comment 5.1**

**Response:** In the revised version, the wording was changed to describe the respondents in more objective demographic terms, focusing on age group, gender distribution, and educational attainment rather than assigning unsupported interpretive labels such as “mature.”

**Comment 5.2**

**Response: **In the revised manuscript, the item tables no longer rely only on x/y codes; instead, the actual item statements are shown in Tables 3 and 4. This was done to make the content of the operational measures more transparent and interpretable to readers.

**Comment 5.3**

**Response:** In the revised version, the analytical emphasis was redirected toward the correlation and regression results, and the manuscript clarifies that the core tested relationships are based on these analyses. The conceptual model was also simplified in Figure 1 to better match the actual analytical design.

**Comment 5.4**

**Response: **We appreciate this methodological concern. In the revised manuscript, the earlier descriptive hypothesis was removed and replaced with two hypotheses that correspond directly to the correlation and regression analyses used in the study. This revision was made to ensure better consistency between the hypotheses and the statistical procedures.

**6. Discussion, Conclusion, and Limitations**

**Comment 6.1**

**Response:** The discussion was substantially expanded in the revised manuscript. The revised text now interprets the main findings in relation to prior literature, discusses the implementation gap between data collection and analytical use, and explains the findings in relation to the Iraqi/Nineveh service context. This was done to move the section beyond a brief summary of results toward a more analytical discussion.

**Comment 6.2**

**Response: **In the revised manuscript, we placed greater emphasis on the contextual value of the study by discussing the digital-service environment in Nineveh Governorate and the broader Iraqi developing context, particularly in relation to digital maturity, service-sector conditions, and local organizational capacity. This contextualization was added in the introduction, the study-importance section, the discussion, and the conclusion.

**Comment 6.3**

**Response: ** The conclusion was rewritten in a more synthetic form. In the revised version, it now includes a theoretical conclusion concerning the strategic and informational significance of digital footprints, a practical conclusion concerning the need to strengthen analytical capability and service design in ISP firms, and a contextual conclusion concerning the relevance of the study in Nineveh Governorate and Iraq.

**Comment 6.4**

**Response: **In response, the revised conclusion now incorporates practical recommendations directed toward ISP managers, including strengthening internal analytical capabilities, improving the connection between digital data and service decisions, and balancing data use with privacy and trust considerations.

**Comment 6.5**

**Response: **The revised limitations section now explicitly states that customer experience was assessed only from the ISP-side perspective and that no customer-side data were collected. In addition, future research directions were added, including dual-perspective ISP/customer studies, cross-governorate comparisons, and models incorporating trust, privacy, and analytics capability as mediating or moderating variables.
